# Advances in Proteomics and Functional Foods from Fermentation and Bioencapsulation of Andean Grains and Tubers: Applications and Perspectives

**DOI:** 10.3390/foods15030425

**Published:** 2026-01-24

**Authors:** Wendy Akemmy Castañeda-Rodríguez, Abel José Rodríguez-Yparraguirre, Carlos Diego Rodríguez-Yparraguirre, Wilson Arcenio Maco-Vásquez, Iván Martín Olivares-Espino, Andrés D. Epifanía-Huerta, Oswaldo Lara-Rivera, Elías Guarniz-Vásquez, César Moreno-Rojo, Elza Aguirre

**Affiliations:** 1 Doctoral Program in Agro-Industrial Engineering, Specialization in Advanced Processing of Andean Grains and Tubers, Universidad Nacional del Santa, Nuevo Chimbote 02712, Ancash, Peru; 2025818022@uns.edu.pe; 2 Department of Agroindustry and Agronomy, Faculty of Engineering, Universidad Nacional del Santa, Nuevo Chimbote 02712, Ancash, Peru; cmoreno@uns.edu.pe (C.M.-R.); eaguirre@uns.edu.pe (E.A.); 3 Graduate School, Universidad Nacional de Trujillo, Trujillo 130101, La Libertad, Peru; cdrodriguezy@unitru.edu.pe (C.D.R.-Y.); wmaco@unitru.edu.pe (W.A.M.-V.); iolivares@unitru.edu.pe (I.M.O.-E.); 4 Graduate School, Universidad Privada de Trujillo, Trujillo 130101, La Libertad, Peru; andres.epifania@uprit.edu.pe; 5 Graduate School, Universidad Nacional de Barranca, Barranca 15312, Lima, Peru; olara@unab.edu.pe; 6 Graduate School, Universidad Privada San Pedro, Chimbote 02803, Ancash, Peru; eguarnizv@usp.edu.pe

**Keywords:** biotransformation, peptides, biotechnology, functional foods, sustainability, pseudocereals

## Abstract

The transformation of Andean grains and tubers through fermentation and bioencapsulation has emerged as a key strategy to enhance their nutritional, functional, and biotechnological value, driven by advances in proteomic and metabolomic techniques. This study aimed to systematize recent evidence on the biochemical and functional modifications induced by these processes and their potential application in the development of functional foods. The methodology integrated 67 studies analyzed using tools such as R 4.5.1 with the JupyterLab interface 4.5.2, SCImago Graphica Beta 1.0.53, and VOSviewer 1.6.20, incorporating data generated through LC-MS/MS, UHPLC-QTOF, Orbitrap platforms, transcriptomics, and combined omics approaches, considering original studies published between 2020 and 2025. The main findings indicate substantial increases in free amino acids (up to 64.8%), phenolic compounds (2.9–5.2%), and antioxidant activity (up to 45%), along with the identification of 430 polyphenols, 90 flavonoids, 14 novel oxindole acetates, and bioactive peptides with IC50 values ranging from 0.51 to 0.78 mg/mL. Bioencapsulation showed controlled release of bioactive compounds, highlighting nanocapsules of 133–165 nm with a maximum release of 9.86 mg GAE/g. In conclusion, the combination of fermentation and encapsulation enhances the stability, bioavailability, and functionality of Andean crops, supporting their industrial adoption for the development of sustainable nutraceutical foods that improve health and promote the valorization of traditional resources.

## 1. Introduction

Global interest in functional foods has increased substantially, driven by the demand for healthier, more sustainable alternatives with higher nutritional density compared to ultra-processed products. In this context, Andean grains and tubers have emerged as strategic matrices due to their high concentrations of proteins, bioactive peptides, vitamins, and secondary metabolites with nutraceutical potential. Recent studies report that food formulations based on orange-fleshed sweet potato, amaranth, soybean, and pumpkin seeds can reach 18.3% protein and exhibit elevated levels of provitamin A carotenoids, clearly surpassing the nutritional density of conventional cereals [1,2]. Traditional crops such as quinoa, amaranth, potato, and mashua are also rich in nutrients and secondary metabolites; however, their bioavailability may be limited by the presence of antinutritional factors [3,4,5,6]. In response, advanced processing strategies, including fermentation and bioencapsulation, have gained increasing attention as effective approaches to enhance proteomic attributes and functional potential by improving nutrient stability and controlled release [7,8]. Collectively, these advances underscore the need to explore innovative strategies that revalorize Andean biodiversity for sustainable, health-oriented applications [9].

Studies on Andean grains and tubers processed through fermentation and emerging technologies show relevant quantitative advances that support the growing interest in their application to functional foods. Reports indicate that the incorporation of *Lactiplantibacillus plantarum* and propionic acid improves the fermentative quality of amaranth silage, resulting in a marked increase in organic acids and a substantial reduction in dry matter losses compared with conventional treatments [10]. Complementarily, bioencapsulation protects sensitive molecules during gastrointestinal transit, ensuring controlled release and improved bioavailability [11]. The various technological approaches have shown notable potential when applied to Andean crops, where proteins and phytochemicals can be preserved and optimized for functional applications [12]. The combination of both processes is considered a cutting-edge strategy for generating innovative products that integrate ancestral biodiversity with modern nutritional science [13].

In the transformation of foods derived from Andean functional matrices, significant gaps still persist in the proteomic and physicochemical characterization of products obtained through these technologies. Across different food-processing approaches, it has been reported that the combined treatment involving fermentation, ultrasound, and hydrocolloids in quinoa-based beverages enhances stability and sensory acceptance; however, the specific protein-level changes responsible for these improvements have not been detailed [12]. Similarly, increases greater than 15% in proteins and bioactive peptides have been reported in pasta formulated with germinated quinoa and amaranth grains, although the complete identification of the generated peptides and their behavior during thermal processing have not yet been elucidated [13]. These gaps hinder process optimization and the design of highly functional foods based on Andean biodiversity, considering that studies have shown that the low bioavailability of polyphenols can be improved through starch encapsulation, achieving efficiencies of 74–79% and stabilizing over 90% of the compound during storage [14].

Traditionally, Andean grains and tubers have been processed through spontaneous fermentations, germination, and prolonged cooking, practices that reduced antinutritional factors and improved palatability but offered limited control over microbiological stability and product reproducibility. Modern controlled fermentation technologies represent a direct evolution of these ancestral processes, enabling regulation of metabolic activity and the targeted generation of functional compounds. Liquid fermentation of quinoa and amaranth flours with *Weissella cibaria* and *Lactobacillus plantarum* achieved protein degradation levels close to 51% and exopolysaccharide production of up to 20.79 g/kg, improving both texture and acid profile [15]. Similarly, submerged fermentations of sweet potato with *Bacillus coagulans* generated up to 49 volatile compounds and the highest total acidity among evaluated strains, demonstrating precise control over fermentative metabolism [16]. In parallel, bioencapsulation technologies have enabled the stabilization and enhancement of these bioactive compounds, achieving encapsulation efficiencies above 95% for amaranth peptides with angiotensin-converting enzyme (ACE) inhibitory activity retained after in vitro digestion [9], and up to 86% for purple potato anthocyanins, with a 20% increase in bioaccessibility [17]. Collectively, the integration of controlled fermentation and bioencapsulation constitutes a technological bridge between traditional Andean knowledge and modern processing systems, enabling the development of reproducible, stable functional foods with targeted functionality.

Moreover, technological development and industrial scale-up face challenges related to microbiological stability and the incorporation of functional agents. Results have shown strong antimicrobial activity, suggesting opportunities to enhance the safety of fermented products, although concerns remain regarding regulatory acceptance and potential environmental impact. The functional beverage industry is challenged with developing plant-based products that exhibit high stability, good sensory acceptability, and consistent nutritional value. In the case of quinoa, one of the main obstacles is the presence of saponins, compounds that negatively affect flavor and limit their inclusion in liquid formulations. Bioprocessing techniques such as soaking, germination, and malting have been shown to drastically reduce these compounds, decreasing levels from 0.06% in raw grains to as low as 0.02%, and even achieving complete removal through soaking [12].

Within this framework, the present study aims to systematize recent advances in fermentation and bioencapsulation applied to Andean grains and tubers, with an emphasis on their proteomic and functional effects, as well as to identify the main challenges limiting their industrial application. A critical review of the literature is proposed to understand the underlying mechanisms, highlight convergences and controversies, and provide perspectives to guide the development of innovative functional foods based on Andean biodiversity.

## 2. Materials and Methods

### 2.1. Eligibility Criteria

The eligibility criteria for this systematic review were defined to ensure scientific rigor, relevance, and comparability of the selected studies. Inclusion criteria comprised peer-reviewed articles published in English that addressed Andean grains and tubers, focusing on fermentation, bioencapsulation, metabolomics, proteomics, or functional food applications, and that reported quantitative outcomes related to nutritional, bioactive, technological, or sensory properties. Studies employing in vitro, in vivo, or advanced analytical approaches were considered eligible. Exclusion criteria included conference abstracts, review papers, non-experimental reports, studies lacking methodological clarity, and works not providing measurable functional or biochemical outcomes. For synthesis purposes, the selected studies were grouped into thematic categories, including microbial fermentation strategies, micro- and nanoencapsulation technologies, metabolomic and proteomic characterization, edible coatings and films, and regulatory or sustainability-related aspects, enabling a structured qualitative and comparative analysis of emerging trends.

### 2.2. Information Sources

The information sources for this systematic review were selected to ensure comprehensive coverage of high-quality scientific literature related to Andean grains and tubers. Electronic databases consulted included Web of Science and Scopus, which were systematically searched for peer-reviewed articles addressing fermentation, bioencapsulation, metabolomics, and functional food applications. Reference lists of selected articles were manually screened to capture further pertinent studies. All databases and resources were last searched and consulted in June 2025, ensuring that the review reflects the most up-to-date evidence available at the time of analysis. A comprehensive literature search was conducted using the Scopus and Web of Science international academic database covering the period from January 2020 to June 2025.

### 2.3. Search Strategy

The search strategy combined descriptors in English and Spanish, employing Boolean operators (“AND”, “OR”) and MeSH terms whenever possible (Table 1). Relevant original articles addressing fermentation, bioencapsulation, proteomics, or functional foods derived from Andean crops (grains and tubers) were included.

The strategy used for document retrieval in the Web of Science database employed the following Boolean code: (“bioencapsulation” OR “microencapsulation” OR “nanoencapsulation” OR “controlled release” OR “delivery systems” OR “encapsulation technologies” OR Alginate) AND (“Andean grains” OR quinoa OR amaranth OR kaniwa OR “Chenopodium quinoa” OR “*Chenopodium pallidicaule*” OR “Andean tubers” OR potato OR “*Solanum tuberosum*” OR oca OR mashua OR ulluco).

For the Scopus database, the following search code was used: (“proteomics” OR “protein profiling” OR “protein characterization” OR “protein expression” OR “protein fingerprinting” OR “protein identification” OR “mass spectrometry” OR “MS-based analysis” OR “LC-MS” OR “LC-MS/MS” OR “shotgun proteomics” OR “quantitative proteomics” OR “label-free proteomics” OR “metaproteomics” OR “food proteomics” OR “nutritional proteomics”) AND (“functional foods” OR “bioactive compounds” OR nutraceuticals OR “health-promoting foods” OR “functional ingredients” OR “functional properties” OR “phytochemical composition” OR fermentation OR “lactic acid fermentation” OR “solid-state fermentation” OR “microbial fermentation” OR “fermented foods” OR “fermented grains” OR “postbiotics” OR “metabolites” OR “bioactive peptides” OR “peptide release” OR “enzymatic hydrolysis” OR “biotransformation”) AND (“Andean grains” OR quinoa OR amaranth OR kaniwa OR “*Chenopodium quinoa*” OR “*Chenopodium pallidicaule*” OR “*Andean tubers*” OR *potato* OR “*Solanum tuberosum*” OR *oca OR* mashua OR ulluco).

### 2.4. Study Selection Process

**Figure 1:**
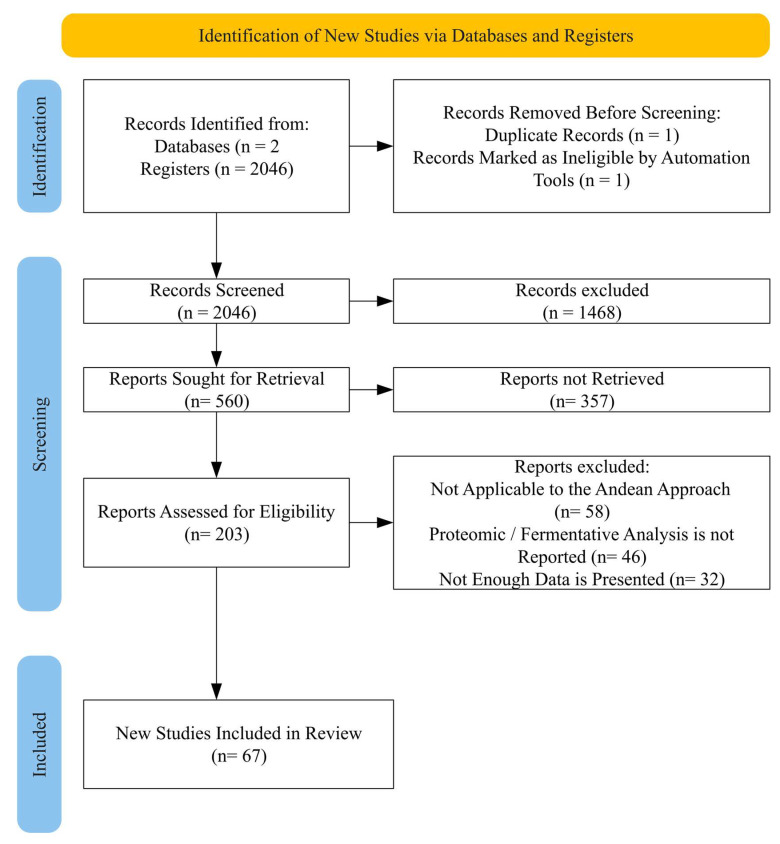
PRISMA Flow Diagram for Document Selection.

The selection process was guided by the PRISMA protocol (https://www.prisma-statement.org/prisma-2020-statement(accessed on 21 November 2025)), encompassing four specific stages (Figure 1): (i) identification of articles using keywords, (ii) screening of titles and abstracts to exclude irrelevant studies, (iii) full-text reading of the selected articles, and (iv) application of predefined inclusion and exclusion criteria. Publications focusing on Andean grains and tubers—preferably quinoa, amaranth, mashua, potato, and oca—were included, provided they were related to enzymatic fermentation techniques, bioencapsulation, or proteomic analysis. Duplicate studies obtained across different search blocks were excluded, as procedures were implemented to remove duplicates due to the overlap of topics on advanced techniques and functional foods. To merge the databases, the R 4.5.1 compiler with the JupyterLab interface 4.5.2 was used, and keyword bibliometric analysis for emerging topics was conducted using VOSviewer 1.6.20 and SCImago Graphica Beta 1.0.53.

### 2.5. Data Extraction Process

The data extraction process was carried out using a standardized, predefined protocol to ensure consistency and reproducibility across all included studies. Reviewers extracted data from each eligible publication, working in parallel to minimize bias and transcription errors. The extracted information included study design, biological matrix, processing or encapsulation technology, analytical techniques employed, key functional outcomes, and main quantitative results. Discrepancies between reviewers were resolved through discussion, and when necessary, the document was comprehensively analyzed. Data were recorded in structured extraction tables developed in Excel spreadsheet format and supported by RStudio 4.5.1 for data organization and preprocessing. Fully automated text mining tools were not used; however, reference management software was employed to organize records and remove duplicates.

### 2.6. Data List

The review systematically collected data across predefined outcome domains related to the biotransformation of Andean grains and tubers. These domains included: (i) metabolomic and proteomic changes (such as phenolics, peptides, saponins, and flavonoids); (ii) biofunctional activities (antioxidant, antihypertensive, antiglycemic, and antimicrobial effects); (iii) technological performance indicators (encapsulation efficiency, particle size, ζ-potential, release kinetics, and stability); and (iv) quality-related parameters (digestibility, sensory attributes, and shelf life). Whenever possible, all compatible outcomes within each domain were extracted across analytical methods, measurement scales, time points, and experimental conditions, prioritizing standardized assays and the most comprehensive datasets. Additional variables captured included the type of raw matrix or species, processing and fermentation technologies, microbial strains or enzymes employed, analytical platforms, and specific experimental conditions.

### 2.7. Synthesis Methods

The synthesis followed a structured and transparent process to ensure consistency across the reviewed evidence. Studies were first grouped according to matrix type (grains or tubers), bioprocessing strategy (fermentation, bioencapsulation, or integrated approaches), and analytical scope (proteomics, metabolomics, or multi-omics), and their key characteristics were tabulated and compared against the predefined synthesis framework. Prior to synthesis, data were harmonized by standardizing units, normalizing quantitative indicators when required, and clearly flagging missing or non-comparable summary statistics without imputation. Results from individual studies were organized into summary tables and thematic figures to facilitate qualitative comparison. Given the high methodological and biological heterogeneity across matrices, technologies, and analytical platforms, a narrative and descriptive synthesis was prioritized over meta-analysis, allowing integration of mechanistic, functional, and technological findings. Potential sources of heterogeneity were explored qualitatively through subgroup comparisons by crop species, processing method, and analytical technique.

## 3. Fermentation of Andean Grains and Tubers

**Figure 2:**
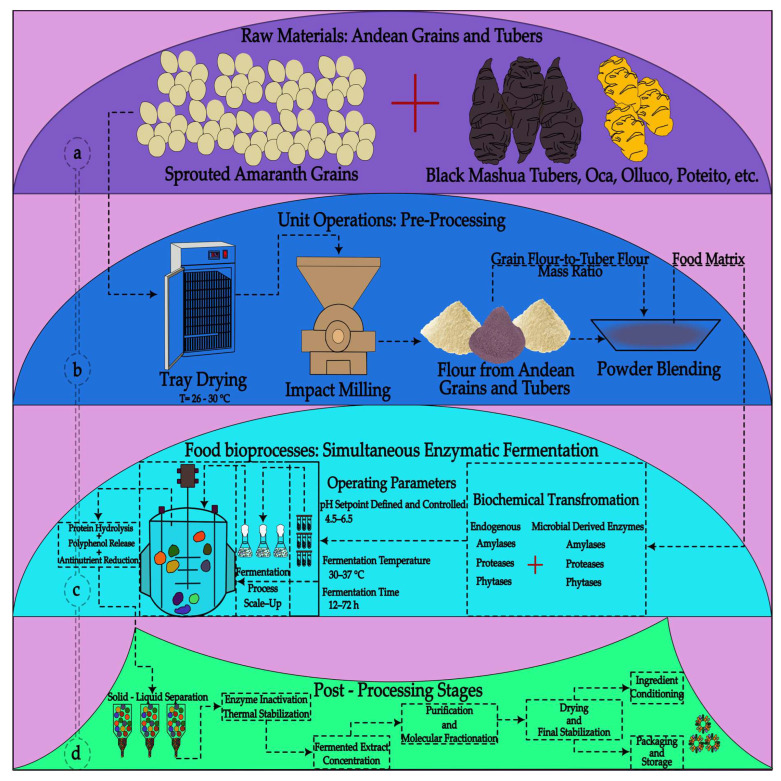
General Fermentation Process in Andean Grains and Tubers. General fermentation-based bioprocessing scheme for Andean grains and tubers. (**a**) Raw material matrix, comprising sprouted amaranth grains and Andean tubers (black mashua, oca, olluco, potato, etc.), selected as complementary carbohydrate–protein sources. (**b**) Pre-processing unit operations, including controlled tray drying (26–30 °C), impact milling and powder blending, where the grain flour–to–tuber flour mass ratio is adjusted to obtain a homogeneous food matrix with tailored physicochemical properties. (**c**) Simultaneous enzymatic fermentation stage, representing the core biochemical transformation step. Endogenous enzymes (amylases, proteases and phytases) act synergistically with microbial-derived enzymes under defined and controlled operating parameters (pH 4.5–6.5, temperature 30–37 °C and fermentation time 12–72 h), promoting protein hydrolysis, phenolic compound release and antinutrient reduction. This stage also highlights the potential for fermentation process scale-up. (**d**) Post-processing stages, including solid–liquid separation, enzyme inactivation and thermal stabilization, concentration of the fermented extract, purification and molecular fractionation, followed by drying, ingredient conditioning, packaging and storage, enabling the production of stable and standardized functional ingredients for food and nutraceutical applications.

Fermentation of Andean grains has emerged as a key technology to enhance their nutritional and functional potential through controlled microbial processes (Figure 2). In species such as quinoa (*Chenopodium quinoa*), amaranth (*Amaranthus caudatus*), and tarwi (*Lupinus mutabilis*), the metabolism of lactic acid bacteria and yeasts leads to the degradation of antinutritional compounds, such as saponins and phytates, while increasing the bioavailability of essential minerals and free amino acids [18]. At the proteomic level, enzymatic proteolysis during fermentation generates low-molecular-weight peptides exhibiting bioactive activities related to blood pressure regulation, modulation of the immune response, and free radical scavenging [19]. This set of transformations positions fermentation as a process capable of revalorizing ancestral crops, integrating functional benefits and organoleptic improvements, which is strategic for the development of innovative and culturally relevant foods in Andean contexts [20].

Likewise, the fermentation of Andean grains and tubers represents a key biotechnological strategy to enhance functionality, release bioactive compounds, and transform technological properties. In protein-rich matrices such as amaranth and quinoa, the use of specific starter cultures has shown remarkable results; for instance, liquid sourdough fermentation with *Weissella cibaria* C43-11 and *Lactobacillus plantarum* ITM21B for 15 h achieved high exopolysaccharide production (approximately 20.79 g/kg at 250 DY), along with nearly 51% protein degradation, promoting improved texture and higher metabolic availability [15]. In tubers such as sweet potato, submerged fermentation with various strains revealed the production of 49 volatile compounds, with *Bacillus coagulans* exhibiting the most diverse ester profile and the highest total acidity, thereby shifting the aromatic character toward fermentative alcohols and aldehydes [16]. Complementarily, solid-state fermentation applied to grain mixtures has shown increases in essential amino acids, enzymatic activities, and antioxidant capacity after 36 h [21]. Meanwhile, in quinoa proteins, 91 bioactive peptides with ACE-inhibitory activity were identified [22].

### 3.1. Enzymatic Fermentation of Andean Grains

Enzymatic fermentation applied to Andean grains, such as quinoa (*Chenopodium quinoa*) and amaranth (*Amaranthus caudatus* and *Amaranthus hypochondriacus*), represents an innovative biotechnological tool to enhance nutrient bioavailability and generate bioactive compounds of functional interest. In this process, the action of microbial proteases and specific hydrolases triggers the hydrolysis of storage proteins, releasing low-molecular-weight peptides with potential antioxidant, antihypertensive, and immune-modulating properties [23]. Additionally, the partial degradation of polysaccharides and starches increases the availability of fermentable sugars, thereby promoting both microbial growth and the production of secondary metabolites associated with health benefits [24]. These enzymatic modifications not only provide functional value but also reduce antinutritional factors and improve the overall digestibility of the grains, making them an ideal platform for the development of functional foods with high nutritional and cultural impact [25].

**Figure 3:**
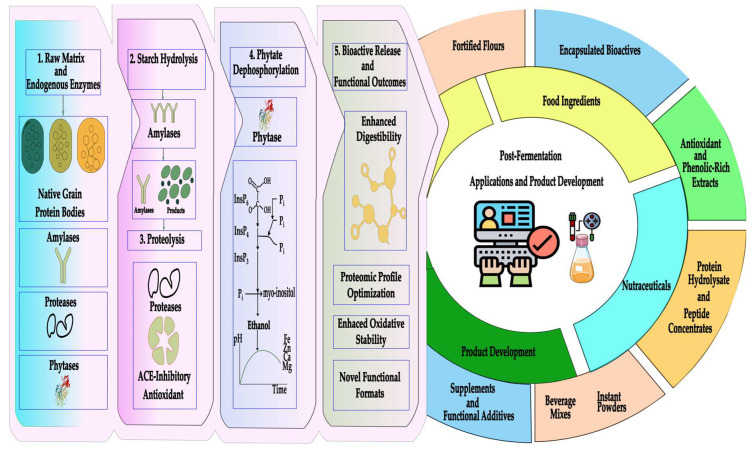
Biotechnological Stages of Fermentation in Andean Grains and Tubers and applications. Schematic representation of the main biotechnological fermentation stages in Andean grains and tubers, highlighting enzymatic hydrolysis, phytate degradation, bioactive release, and their translation into functional food ingredients and nutraceutical applications.

The fermentation process applied to Andean matrices continues to reveal significant effects on the stability, functionality, and bioactivity of their protein and phenolic components (Figure 3). In sweet potato used for protein gel formation, prior controlled fermentation has been shown to enhance color stability and improve anthocyanin retention against oxidative processes, achieving a remarkable efficiency of 87.27%, highlighting its potential as a highly stable functional ingredient [26]. In amaranth proteins, fermentation processes mediated by *Lactobacillus* spp. promote the release of bioactive peptides, including enzyme inhibitors with IC_50_ values around 0.47 mg/mL, as well as peptide profiles associated with antioxidant and antihypertensive pathways, thereby expanding their nutraceutical applicability [27]. An alternative approach is observed in the fermentation of sweet potato, soybean, and agro-industrial residues using *Ganoderma lucidum*, where substantial increases in antioxidant compounds were recorded, reaching 11.43, 32.64, and 40.19 μmol Trolox/100 g, along with bioactive polysaccharides increasing to 19.29, 17.70, and 32.35 μmol Trolox/100 g depending on the substrate [28]. Additionally, germination followed by co-fermentation in buckwheat and quinoa improves the phenolic profile, increases antioxidants, and reduces antinutrients, achieving reductions in tannins of 83% and 20% through germination, respectively [29].

Proteomic studies of enzymatic fermentation in quinoa and amaranth (*Amaranthus caudatus* and *Amaranthus hypochondriacus*) have enabled more precise mapping of structural changes in protein fractions during the process. Additionally, analysis using high-resolution mass spectrometry techniques has identified peptides derived from enzymatic hydrolysis with bioactive sequences previously described using in vitro models [23]. These compounds exhibit the ability to inhibit key enzymes, such as angiotensin-converting enzyme (ACE), and display antioxidant properties associated with the reduction of oxidative stress [30]. Furthermore, it has been observed that combining enzymatic fermentation with lactic acid microorganisms enhances the generation of bioactive metabolites and imparts a more appealing sensory profile to the products [31]. In this way, the integration of enzymatic and proteomic techniques opens new possibilities for the systematic identification of functional biomarkers in fermented Andean grains (Table 2), achieving an essential combination in the transformation of Andean matrices.

### 3.2. Andean Matrices Based on Traditional and Non-Traditional Tubers

Enzymatic fermentation applied to Andean tubers, such as potato (*Solanum tuberosum*), mashua (*Tropaeolum tuberosum*), and oca (*Oxalis tuberosa*), represents an innovative strategy to modify the chemical matrix and enhance their functional profile. In these crops, microbial enzymes (mainly amylases, proteases, and glucosidases) promote the hydrolysis of starches and proteins, leading to the release of fermentable sugars and low-molecular-weight bioactive peptides. This process facilitates the reduction of antinutritional compounds, improves the bioavailability of essential micronutrients, and converts complex carbohydrates into more readily assimilable metabolites [35]. Furthermore, enzymatic fermentation in tubers has been associated with the generation of organic acids, exopolysaccharides, and free phenolic compounds, which enhance the antioxidant capacity and prebiotic functionality of the resulting products. This positions fermented tubers as a relevant source of functional ingredients for the development of healthy and sustainable foods [23].

Fermentation has demonstrated differentiated effects when applied to non-traditional matrices and processes inspired by Asian technologies, providing relevant insights for the valorization of Andean grains and tubers. In fermentation systems such as moromi and shochu, temperature modulation governs lactic acid production kinetics and enzymatic activity, resulting in distinct sensory profiles. Moromi fermented at 38 °C shows higher amino acid accumulation, whereas shochu produced at 25 °C exhibits enhanced fruity notes associated with the formation of volatile esters. In parallel, targeted fermentation strategies employing specialized fungi for saponin degradation constitute an effective approach to mitigate the characteristic bitterness of several Andean crops. Reported optimal parameters, including a potato concentration of 97.3 mg/mL, glucose at 20.6 mg/mL, pH 2.1, and a temperature of 29.2 °C, over 6 days, maximize desirable secondary metabolites while reducing antinutritional compounds. Furthermore, tubers such as olluco and arracacha exhibit increased phenolic content and antioxidant capacity following fermentation, with dietary fiber contents of 14% in olluco and 4.07% in arracacha. Collectively, these findings highlight fermentation as versatile [34].

## 4. Bioencapsulation in Andean Functional Foods

### 4.1. Microencapsulation and Nanoencapsulation Strategies

**Figure 4:**
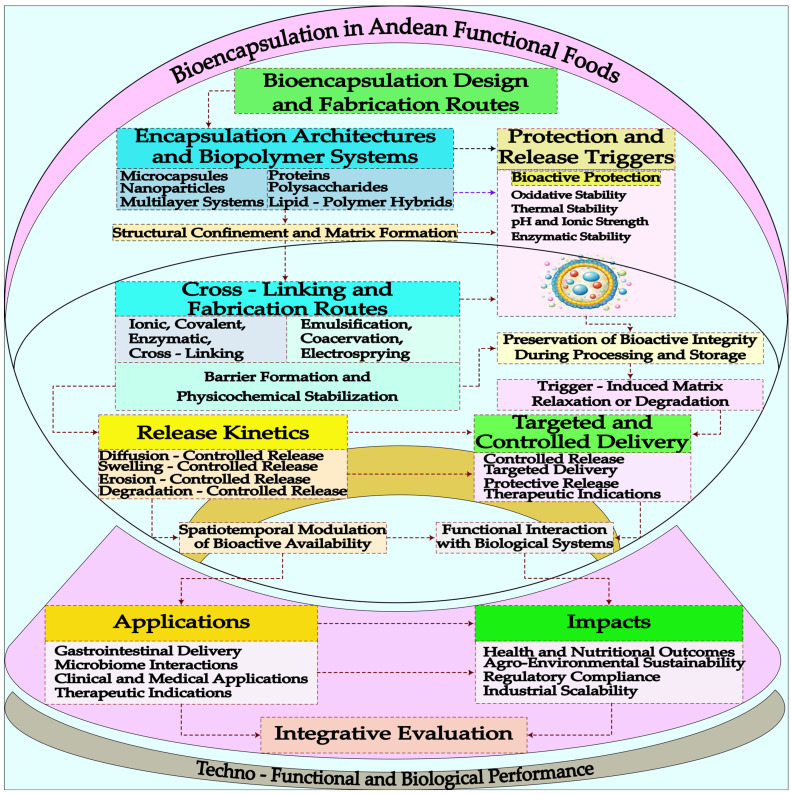
General Mechanism of Bioactive Compound Protection and Release through Bioencapsulation and applications. The three compounds represent Applications, Technologies, and Impacts, interconnected through directional arrows indicating a sequential and feedback-driven process. Bioencapsulation enables protective, controlled, and targeted delivery, supporting applications such as gastrointestinal delivery and microbiome interactions, as well as clinical and therapeutic indications. Technological components include encapsulation architectures, biopolymer-based systems, cross-linking and fabrication routes, and mechanistically defined release triggers (pH variation, ionic strength, and enzymatic activity), governed by kinetic release models. These integrated processes lead to measurable impacts on health outcomes, agro-environmental sustainability, and integrative system evaluation. Arrows denote the dynamic coupling between technology design, functional performance, and system-level impacts.

Bioencapsulation of bioactive compounds derived from Andean grains and tubers constitutes a fundamental tool to ensure their stability, bioavailability, and controlled release within food matrices (Figure 4). Likewise, microencapsulation using techniques such as spray drying, complex coacervation, or ionic gelation has proven effective in protecting antioxidants, peptides, and phenolic compounds against adverse processing and storage conditions [23]. In combined systems of carrot phenolic extract and potato protein, protein–pectin coacervates generate particles ranging from 65.05 to 152.47 µm, increasing encapsulation efficiency up to 69.26–90.15% and reducing apparent retention upon emulsification [36]. High-pressure technologies also show remarkable results: starch hydrogels obtained at 600 MPa for 15 min maintained stable gelatinization and enabled controlled release of polyphenols, as confirmed by FT-IR analysis and Franz-type diffusion assays. [37]. Similarly, amaranth hydrolysates encapsulated in alginate–pectin beads achieved 95.57% encapsulation efficiency and an ACE-inhibitory activity of 97.97%, which was retained even after in vitro digestion [9]. Finally, protein matrices from soybean or potato combined with starch produced beads measuring 2.18–2.64 mm with encapsulation efficiencies of 70.93–82.59%, where 50% IPS–50% starch formulations provided superior protection of carotenoids, extending their shelf life up to 106 days [38].

In native potato, nanoencapsulation of phenolic extracts using spray-drying and nanoprecipitation under optimized conditions (120 °C, 141 L/h) produced nanoparticles of 133–165 nm with negative ζ-potential, low water activity, high encapsulation efficiency, and a maximum phenolic release of 9.86 mg GAE/g, significantly enhancing antioxidant activity measured by DPPH assays [39]. Similarly, quinoa protein hydrolysates formulated through micro- and nanoencapsulation showed strong inhibitory activity against cholesterol esterase (CI_50_ = 0.51 mg/mL) and pancreatic lipase (CI_50_ = 0.78 mg/mL), with 4–12 active peptides identified, supporting their application in cardiometabolic health [40]. Yeast-based microencapsulation combined with polysaccharide matrices further improved anthocyanin retention and release under hydrolytic conditions [41]. Collectively, these strategies position microencapsulation and nanoencapsulation as enabling platforms for the valorization of Andean crops, facilitating the development of functional foods, active packaging, and biotechnological applications aligned with sustainability and health-oriented food systems.

Microencapsulation and nanoencapsulation strategies have emerged as pivotal technologies in the development of Andean functional foods, enabling the stabilization, controlled release, and technological integration of bioactive compounds into complex matrices. In potato-based systems, alginate–biopolymer microcapsules achieved high encapsulation efficiency (87%) and, when incorporated into composite films, conferred up to 46% antioxidant activity, enhanced UV barrier properties, and extended the postharvest freshness of blueberries, demonstrating their applicability in active packaging [42]. Beyond food matrices, alginate–chitosan microcapsules loaded with methyl jasmonate allowed effective postharvest regulation at reduced doses, highlighting the efficiency of encapsulated delivery systems [43]. Encapsulation of functional oils within hydrocolloid matrices improved thermal diffusivity, reduced baking time, and enhanced porosity and lipid profiles in gluten-free products, underscoring industrial productivity gains [44]. At the biological interface, alginate-based encapsulation optimized potato micropropagation, achieving near-complete conversion and rapid shoot development [45]. Complementarily, active films derived from amaranth proteins and hydrolysates exhibited tunable mechanical properties depending on plasticizer and phenolic content [46], while nanoencapsulation of potato phenolics via spray-based systems maximized retention and antioxidant capacity under optimized thermal conditions [47].

Likewise, another relevant aspect of bioencapsulation is the ability to design systems with targeted and controlled release, directed to specific organs or functions in the human body (Table 3). Encapsulation of antihypertensive peptides obtained from enzymatic fermentation of quinoa and tarwi in alginate–chitosan matrices allow their gradual release in the gastrointestinal tract, enhancing their therapeutic effect. In the case of quinoa, protein-based emulsion gels showed significant improvements in functional parameters: Quinoa Protein Hydrolysate (QPH) increased the S_0_ modulus (*p* = 0.006) and emulsifying activity (*p* = 0.002), although system stability decreased (*p* < 0.000), maintaining a water-holding capacity close to 70%, while concentrations between 0.5–2.0% allowed the formation of well-defined three-dimensional networks [48]. Additionally, the encapsulation of compounds extracted from sweet potato using ionotropic alginate beads achieved 60% carotenoid retention and 61–64% phenolic retention, with controlled losses during 60 days of storage (retention of 43–59%) and degradation kinetics of k = 0.0149–0.0106 d^−1^ [49].

Similarly, the incorporation of antioxidants encapsulated in lipid nanoparticles enhances their protection against gastric pH and ensures their availability in the small intestine. These intelligent delivery systems can contribute to the formulation of personalized foods, aligned with the precision nutrition trend, opening a field of great interest for both science and the Andean functional food industry. In nano-structured formulations, ferulic acid encapsulated with quinoa proteins and zein showed efficiencies of 81.2% and 70.7%, respectively, with higher loading in quinoa (29.7%) and greater gastric resistance, optimizing intestinal release [51]. Finally, anthocyanins from purple potato microencapsulated by spray-drying using quinoa starch and gum Arabic achieved 86% efficiency, reduced degradation, and increased bioaccessibility by 20% during digestion [17].

### 4.2. Controlled-Release Systems of Bioactive Compounds

Controlled-release systems of bioactive compounds represent one of the most significant areas of interest in the development of functional foods, as they allow modulation of the rate, site of absorption, and bioavailability of nutritionally relevant molecules. In Andean grains, the incorporation of antioxidant and antihypertensive peptides obtained through fermentation into sustained-release encapsulation systems has been shown to improve their stability during gastrointestinal transit. In tubers such as mashua and potato, which are rich in phenolic compounds and bioactive alkaloids, controlled release through polymeric matrices prevents premature degradation, ensuring delivery to the small intestine at effective concentrations. Edible coatings applied to Andean tubers have emerged as an effective tool to preserve postharvest quality and enhance functional attributes. In fresh potatoes, formulations based on alginate, essential oils, and chitosan significantly improved colorimetric parameters: treatments F1 and F2 increased chroma, while formulations F1–F4 elevated anthocyanin content after three months of storage (*p* < 0.05), maintaining more stable color, higher brightness, and superior sensory acceptability, especially in matrices with higher alginate concentrations [55].

### 4.3. Stability and Bioavailability of Encapsulated Metabolites

The stability of bioactive metabolites represents a central challenge in the development of functional foods, particularly for compounds sensitive to oxygen, light, moisture, or pH. In the case of Andean grains and tubers, molecules such as anthocyanins from native potatoes, flavonoids from quinoa, and carotenoids from oca are highly susceptible to degradation during processing and storage. Encapsulation, using micro- and nanoscale techniques, has proven effective in protecting these metabolites, reducing oxidative losses and extending the shelf life of the products. In parallel, the development of composite films incorporating microcapsules made from alginate–biopolymer systems demonstrated an encapsulation efficiency of 87%, providing a functionally active film matrix. Films containing 4.5% lycopene microcapsules exhibited 46% antioxidant activity, blocked UV radiation, and prolonged the freshness of fruits such as blueberries, highlighting their protective capacity and potential for integration into value chains requiring extended preservation and structural stability [42].

### 4.4. Comparative Assessment of Encapsulating Materials for Andean Functional Foods

The selection of encapsulating materials critically determines the effectiveness of bioencapsulation strategies applied to Andean functional foods, influencing encapsulation efficiency, physicochemical stability, release kinetics, and industrial feasibility. Alginate-based systems are among the most widely used due to their biocompatibility, mild ionic gelation, and strong protective capacity under gastric conditions. Alginate–pectin beads developed for amaranth-derived bioactive peptides achieved encapsulation efficiencies of up to 95.57%, while preserving 97.97% of ACE-inhibitory activity after simulated gastrointestinal digestion, demonstrating their suitability for stabilizing peptide-based nutraceuticals [9]. Similarly, alginate-based beads and coatings applied to sweet potato extracts retained 60% of carotenoids and 61–64% of phenolic compounds, with retention rates of 43–59% after 60 days of storage, although degradation kinetics (k = 0.0106–0.0149 d^−1^) indicated sensitivity to prolonged exposure and environmental stress [49]. Despite these advantages, alginate systems may exhibit limited mechanical strength and susceptibility to high ionic strength or extreme pH, which can compromise matrix integrity during processing or storage [50].

Pectin-based systems, particularly when combined with proteins in coacervate or hybrid matrices, offer improved mechanical stability and enhanced control over release behavior. Protein–pectin coacervates derived from potato protein and carrot phenolic extracts showed particle sizes ranging from 65.05 to 152.47 µm and encapsulation efficiencies between 69.26% and 90.15%, supporting their effectiveness for phenolic protection [36]. However, apparent retention varied widely (53.90–102.16%), highlighting the influence of emulsification conditions and the degree of esterification of pectin on system performance, which may limit reproducibility at industrial scale. Starch and modified starches represent cost-effective and regionally relevant alternatives, particularly for Andean crops. Spray-dried systems using quinoa or potato starch matrices achieved encapsulation efficiencies of approximately 86% for anthocyanins, reducing degradation and increasing bioaccessibility by nearly 20% during digestion [17]. Additionally, starch–gum combinations, such as quinoa starch with Tara gum, enabled the encapsulation of phenolic-rich extracts with contents of 9.60 mg GAE/g and antioxidant capacities of 142.43 µmol/g DPPH, while enabling controlled release of bioactive compounds [39].

Modified Andean starches (OSA) further enhanced encapsulation efficiency, antioxidant retention, and hygroscopic stability in mashua extracts [40]. Nevertheless, starch-based matrices may exhibit lower resistance to enzymatic hydrolysis and variability associated with botanical origin and processing conditions, which can affect long-term stability [37]. Overall, the comparative analysis indicates that no single encapsulating material fully satisfies all technological requirements. Alginate offers superior protection and biocompatibility, pectin enhances structural integrity and release control, and starch-based systems provide economic and regional advantages. Consequently, hybrid or composite encapsulation strategies emerge as the most promising approach to balance stability, controlled release, and scalability in Andean functional food applications [38,42,49].

## 5. Proteomic Advances in Fermented Grains and Tubers

The application of proteomics in the study of fermented Andean grains and tubers has enabled the identification of significant changes in protein composition and functionality (Table 4) following the fermentation process [59]. In grains such as quinoa, amaranth, and cañihua, enzymatic fermentation promotes the partial hydrolysis of storage proteins, generating bioactive peptides with antioxidant, antihypertensive, and immunomodulatory properties [60]. Recent studies on Andean crops have expanded the understanding of the biochemical and molecular mechanisms associated with their quality, resilience, and functional value. In potato, analysis of the Snakin-2 gene revealed contrasting responses between silenced lines (RNAi7) and overexpressed lines (OE27), where the former exhibited 5- to 10-fold increases in COMT and CAD, along with strong induction of Prx10, highlighting a significant reprogramming of lignification and defense pathways. Associated enzymatic activities, including peroxidases, COMT, and CAD, were also elevated, confirming the regulatory role of StSN2 and its interactions with proteins such as Prx2, Prx9, and Prx10 [61].

Likewise, one of the main contributions of proteomics has been the characterization of fermentation-derived peptides with potential therapeutic effects. In studies with fermented quinoa, peptides exhibiting angiotensin-converting enzyme (ACE) inhibitory activity have been identified, opening possibilities for the development of functional foods with antihypertensive effects [83]. Similarly, the fermentation of amaranth has been shown to release peptide sequences with antioxidant properties and the ability to modulate glycemic responses [84]. Under field conditions, potato hybrids analyzed through an integrated proteomic and transcriptomic approach exhibited elevated levels of α-solanine and α-solamargine, accompanied by duplication of the SBT1.7 protease and a marked increase in subtilisin protease. Concurrently, carbonic anhydrase and miraculin were reduced, while endo-1,3-β-glucanase increased 47.96-fold, highlighting its prominent role in metabolic regulation and defense responses [79].

In the case of mashua, proteomic advances have enabled the correlation of bioactive peptide release with antimicrobial properties, which can support the design of natural ingredients for food preservation [85]. These findings reinforce the role of fermentation as a biotechnological strategy to enhance the functionality of native proteins in Andean crops. In the case of amaranth, metabolomic profiling revealed that the LS7 line exhibited the highest accumulation of bioactive compounds, with 2–3-fold increases in provitamin A, elevated vitamin C concentrations, and high levels of total phenolics and flavonoids. This composition translated into superior antioxidant capacity in DPPH and ABTS assays compared to other lines, with LS9 showing slightly lower values. These data highlight the potential of amaranth as a prominent nutraceutical source and underscore the relevance of metabolomics in identifying genotypes with distinct functional profiles [80].

### 5.1. Identification of Bioactive Peptides

The identification of bioactive peptides in fermented grains and tubers requires a rigorous experimental workflow, beginning with optimized sample preparation to preserve endogenous peptides. Peptidomic analyses of the extracts further demonstrated that the fraction adjusted to pH 2 reached 2451 U/mL of protease activity and exhibited strong antibacterial, antifungal, and anticancer effects, with notable activity against A549 and HeLa cell lines, confirming the bioactive and biotechnological potential of peptides derived from quinoa [71]. Peptidomic analysis is performed using nano-LC coupled to high-resolution mass spectrometers (Orbitrap, Q-TOF) or by MALDI-TOF for rapid profiling. It is recommended to combine DDA and DIA (SWATH) acquisition strategies to maximize coverage and quantification of low-abundance peptides [86]. Additionally, the use of isotopically labeled internal controls and synthetic standards enables the assessment of recovery and correction of analytical biases, which are critical aspects when working with complex food matrices such as quinoa or native potato [35].

### 5.2. Enzymatic Proteomics and Protein Digestibility

Enzymatic proteomics applied to fermented Andean grains and tubers has enabled a better understanding of how fermentative processes influence protein degradation and the generation of bioactive peptides [87]. During fermentation, microbial enzymes such as proteases, peptidases, and glycosidases act on storage proteins, reducing their molecular size and enhancing their solubility. In quinoa and amaranth, controlled hydrolysis has been documented, increasing the peptide fraction with antioxidant and antihypertensive properties [64]. In tubers such as potato and mashua, proteins subjected to fermentation exhibit increased susceptibility to enzymatic action, facilitating their subsequent digestion in the gastrointestinal tract [86]. In the Andean grain quinoa, metabolomic studies have enabled precise characterization of the chemical and functional variability of this crop. A comprehensive analysis conducted on 114 accessions reported saponin levels ranging from 0.22 to 15.04 mg/g, with approximately 75% showing low concentrations. Furthermore, twelve oleanane-type saponins and one novel compound were identified, highlighting the breadth and complexity of the saponin profile in the species and its value for the selection and breeding of lines with optimized organoleptic and nutraceutical properties [68].

### 5.3. Relationship Between Proteomics and Functional Properties

Proteomics has enabled the establishment of direct links between changes in the protein profile of fermented Andean grains and tubers and their functional properties [88]. Through mass spectrometry analysis, peptides derived from quinoa and amaranth with antioxidant and antihypertensive capacities have been identified, generated by the action of microbial enzymes during fermentation [89]. In native potato and oca, fermentation leads to the release of partially hydrolyzed proteins that exhibit increased solubility and bioavailability [90]. These protein changes not only enhance digestibility but also increase the presence of bioactive peptides that can exert specific effects on human health, such as glucose modulation and the reduction of oxidative stress [91]. In this regard, an integrated approach combining metabolomics and transcriptomics in seeds and seedlings allowed the detection of 1060 metabolites and 13,095 differentially expressed genes. Among these, lipids and flavonoids were the predominant groups, highlighting pathways related to hormonal signaling and the involvement of AP2/ERF factors as part of the molecular response to high-humidity conditions, a finding that is particularly relevant for understanding the adaptive plasticity of quinoa [69].

### 5.4. Analytical Limitations and Pathway-Oriented Interpretation of Bioactive Peptides

Despite the increasing application of high-resolution proteomic and multi-omics platforms to Andean grains and tubers, the detection of low-abundance bioactive peptides remains a major analytical challenge. Complex food matrices such as quinoa, potato, and amaranth are characterized by a high dynamic range of proteins, secondary metabolites, pigments, and saponins, which can suppress ionization efficiency and reduce peptide detectability during LC–MS/MS analysis. Although advanced metabolomic and integrated omics approaches have enabled the identification of hundreds to thousands of compounds—such as 689 metabolites in colored quinoa varieties, including flavonoids and betacyanins [73], or 1060 metabolites linked to more than 13,000 differentially expressed genes under environmental stress [68]—these datasets often emphasize abundant molecular classes while underrepresenting low-intensity peptide signals. Even in peptidomic studies where strong bioactivities are reported, such as quinoa extracts exhibiting protease activities up to 2451 U/mL with antimicrobial and anticancer effects [70], the specific peptides responsible for these functions are frequently difficult to resolve within untargeted workflows.

As a result, correlations between proteomic profiles and functional properties are frequently derived from global compositional changes rather than from mechanistic, pathway-level evidence. This limitation is evident in multi-omics studies where hundreds of metabolites and genes are identified, yet only a small fraction can be directly linked to functional outcomes. For instance, integrated metabolomic–transcriptomic analyses in purple-fleshed potato identified 18 anthocyanins associated with the differential expression of 12 biosynthetic genes, with St5GT showing marked overexpression as a key determinant of pigment accumulation [63]. Similarly, comprehensive omics profiling across quinoa color variants detected approximately 90 flavonoids and identified 25 regulatory genes, of which only 18 metabolites were classified as functionally discriminant within the flavonoid biosynthesis pathway [77]. These data highlight the need for pathway-oriented interpretation strategies that combine targeted proteomics with metabolomic and transcriptomic resolution. Mapping low-abundance peptides and proteins to specific metabolic routes involved in antioxidant defense, antihypertensive regulation, or inflammatory modulation would enable a more precise functional attribution.

## 6. Food Applications of Fermented and Bioencapsulated Products

The food applications of fermented and bioencapsulated products have gained increasing relevance due to their ability to enhance the nutritional and functional quality of various foods (Table 5). Fermentation processes allow the transformation of raw materials into more stable and safe matrices, enriched with bioactive compounds of higher bioavailability. In parallel, bioencapsulation provides an effective strategy to protect beneficial microorganisms, antioxidants, and labile metabolites, facilitating their incorporation into beverages, dairy products, plant-based derivatives, and fortified snacks. In red quinoa, biochemical studies have highlighted its remarkable α-glucosidase inhibitory capacity, a key mechanism for modulating glycemic response. The purified phenolic extract (BPE) exhibited an IC_50_ of 10.295 mg/mL, accompanied by high antioxidant activity measured by DPPH and ABTS assays. Moreover, it demonstrated relevant functional effects, such as delaying starch digestion and reducing postprandial glucose in experimental models at a dose of 50 mg/kg, underscoring its potential as a nutraceutical ingredient for glycemic control and metabolic disorder management [92].

### 6.1. Development of Functional Foods and Nutraceuticals

The development of functional foods from the fermentation of Andean grains and tubers has become an innovative strategy to harness their bioactive properties [101]. Fermented quinoa and amaranth have been shown to generate peptides with antihypertensive, hypoglycemic, and antioxidant effects, facilitating their incorporation into fermented dairy beverages, cookies, and protein supplements [102]. In the case of potato and mashua, fermentation enhances the release of phenolic compounds and hydrolyzed proteins with anti-inflammatory activity [103]. These foods not only contribute to basic nutrition, but also provide additional benefits that support the prevention of chronic non-communicable diseases, in line with the global trend towards healthier diets, considering the large contribution of proteins, fibers and carbohydrates essential for health [104].

Andean grains and tubers have been widely studied as functional foods due to the bioactivity demonstrated within their food matrix. Red quinoa exhibits significant α-glucosidase inhibition (IC_50_ = 10.295 mg/mL) and high antioxidant capacity as assessed by DPPH and ABTS assays, which translates into slower starch digestion and a significant reduction in postprandial glucose levels in animal models at doses of 50 mg/kg [92]. In amaranth, metabolomic analyses have identified phenolic acids such as caffeic and glucaric acids, whose content can increase by up to 5.2% after cooking; however, intensive domestic processing reduces total polyphenols by 22–60% [93]. In tubers, sweet potato contains more than 4400 secondary metabolites, including flavonoids and phenolic acids shared among varieties [95], while fermentation increases amino acid content by up to 64.83% and enhances phenolic compounds, thereby strengthening its dietary functionality [16].

The nutraceutical potential of Andean grains and tubers is based on the concentration and stabilization of specific bioactive compounds. In quinoa, peptides obtained through chymotrypsin hydrolysis showed CI_50_ values of 0.51 mg/mL for cholesterol esterase (CEase) and 0.78 mg/mL for pancreatic lipase (PL), with 16 inhibitory peptides identified and linked to lipid metabolism modulation [41]. In potato, nanoencapsulated phenolic extracts achieved particle sizes of 133–165 nm and a maximum release of 9.86 mg GAE/g, significantly improving their stability and antioxidant activity [39]. Likewise, sweet potato derived peptides obtained by ultrasound assisted hydrolysis (<3 kDa) exhibited high antioxidant capacity, strong Fe^2+^ chelation, and elevated ORAC values [99]. Collectively, these findings support the development of standardized nutraceuticals derived from Andean matrices, offering targeted applications and enhanced biological efficacy compared with conventional functional foods.

### 6.2. Use in Traditional and Modern Food Matrices

The fermentation of Andean grains and tubers has enabled the enrichment of traditional food matrices such as regional beverages, soups, and breads [60]. In the case of quinoa and kiwicha chicha, fermentation not only enhances their sensory properties but also increases the content of bioactive peptides and antioxidant compounds. Similarly, fermented potato, used in traditional preparations such as “tunta,” has been shown to improve its nutritional profile by enhancing the bioavailability of minerals such as iron and zinc [105]. These practices illustrate how modern biotechnology can support the preservation of traditional knowledge while simultaneously enhancing its health benefits [106]. Such physiological instability poses challenges for maintaining consistent metabolic activity and productivity under the fluctuating conditions typical of continuous or semi-continuous industrial fermentations. Additional constraints arise from disparities in aeration, heat transfer, and reactor configuration between pilot and industrial systems, which can alter metabolic efficiency and final metabolite profiles [16,94].

### 6.3. Implications for the Healthy Food Industry

The incorporation of fermented and bioencapsulated Andean grains and tubers into the healthy food industry represents a strategic opportunity to diversify the range of functional products. Global interest in foods that provide benefits beyond basic nutrition has driven the demand for ingredients with antioxidant, antihypertensive, and glucose-regulating properties [107]. In this context, crops such as fermented quinoa, kiwicha, and potato are positioned as sustainable, high-quality sources for the development of products aimed at nutritionally conscious consumers [46]. Furthermore, their Andean origin adds cultural and biodiversity value, enhancing their appeal in international markets due to their nutritional content and bioactive compounds [108].

On the other hand, the application of ultrasound combined with enzymatic hydrolysis in sweet potato allowed the production of peptides under 3 kDa with high antioxidant capacity, Fe^2+^-chelating activity, OH radical scavenging, and elevated ORAC values. These types of ingredients are highly attractive for the formulation of beverages, bars, and functional supplements, particularly within the growing segment of plant-based protein products. The production of bioactive peptides using clean technologies such as sonication provides a scalable tool aligned with consumer demand for natural products with demonstrable benefits [99]. Likewise, encapsulation studies in tubers—such as sweet potato nodal segments treated with 4% alginate, 100 mM CaCl_2_, and ½ MS—demonstrated improvements in shoot formation, root development, and genetic conservation. Although this technology is closer to the biotechnological field, it has direct implications for the sustainable production of functional raw materials, ensuring uniformity, stability, and reduced losses throughout the production chain [45]. This can translate into improved availability of Andean crops for the healthy food industry, particularly in applications that require stable and standardized raw materials.

## 7. Limitations and Future Perspectives

### 7.1. Technological Challenges in Fermentation and Bioencapsulation of Grains and Tubers

**Figure 5:**
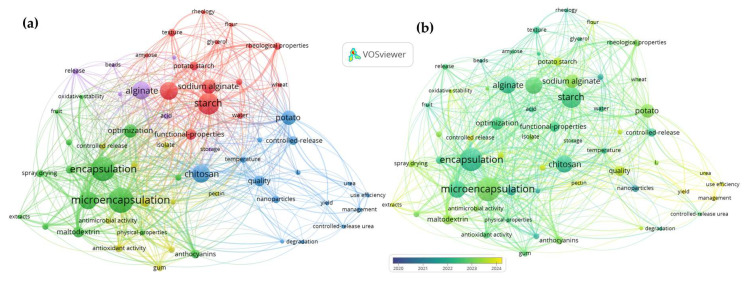
Network visualization of keyword co-occurrence generated with VOSviewer (overlay visualization) for advances in bioencapsulation of Andean grains and tubers. (**a**) Network structure of keyword co-occurrence; (**b**) Overlay visualization showing temporal or density-based color gradients.

One of the main challenges in the fermentation of Andean grains and tubers lies in optimizing microbiological and enzymatic conditions to ensure the consistent production of bioactive compounds [109]. Factors such as pH, temperature, and substrate concentration significantly affect the viability of probiotic strains and the release of functional metabolites. However, fermentative systems applied to starch-rich matrices, such as potato or mashua, exhibit variations in enzymatic hydrolysis rates, which hinder the uniformity of the final product. These limitations are further exacerbated when scaling processes to an industrial level, as optimal conditions observed in the laboratory are not always replicated in larger production facilities. In the field of bioencapsulation, the use of alginate has proven to be an effective strategy (Figure 5) for protecting bioactive compounds during processing and storage. Nonetheless, technological challenges include the need to control microcapsule size and uniformity, as these parameters are critical in determining the controlled release of metabolites [110].

### 7.2. Limitations in the Industrial Scalability of Bioprocesses Applied to Andean Matrices

The industrial scalability of bioprocesses applied to Andean grains and tubers faces structural limitations that extend beyond the microbiological performance demonstrated at the laboratory level. Although various studies have shown high biochemical potential through controlled fermentation, advanced metabolomics, and functional encapsulation, the reproducibility of these results at an industrial scale remains constrained by the intrinsic variability of the raw materials. In this context, quinoa exhibits wide differences in saponin profiles (0.22–15.04 mg/g) among accessions [67], and potato and amaranth cultivars exhibit significant nutraceutical fluctuations depending on color, phenological stage, or growing conditions [63,72,80]. These variations make it challenging to standardize stable production flows, as heterogeneity in moisture content, bulk density, resistant starch levels, pigment composition, and dietary fiber directly affects mass transfer, substrate availability, and microbial stability in large-scale bioreactors [60]. Metabolomic studies in sweet potato and potato have demonstrated substantial variability in secondary metabolites, including flavonoids, phenolic acids, amino acids, and organic acids across varieties and processing conditions, which directly influences fermentation performance and metabolic outcomes [65,95]. Furthermore, post-fermentation studies in sweet potato have shown that changes in pH, sugar depletion, amino acid enrichment, and phenolic accumulation are highly dependent on processing parameters and microbial strains, complicating process reproducibility at scale [16]. In addition, differences in equipment geometry, aeration rates, and thermal distribution between pilot and industrial scale systems often fail to replicate homogeneous fermentation environments, leading to reduced metabolic efficiency and altered synthesis of target metabolites [95,102].

Similarly, at the technological level, many of the reported advances rely on high-precision instrumentation—UHPLC-QTOF, Orbitrap, iTRAQ, and LC-MS/MS—whose costs limit continuous production, especially in rural regions where these crops are concentrated. Fermentation processes using specific strains such as *Aspergillus niger* or *Bacillus coagulans* require highly controlled conditions that are not always reproducible at large-scale plants [16]. Likewise, micro- and nanoencapsulation techniques, while enabling controlled release and stable particles of 133–165 nm [40], still exhibit low coacervation yields (35–48%) and high energy costs, affecting their commercial feasibility. The transition to continuous or semi-continuous systems demands highly stable micro-organisms; however, many native strains exhibit limited tolerance to acidity, osmotic stress, and thermal fluctuations characteristic of industrial operations. Fermentation studies using sweet potato matrices have shown pronounced variations in pH (3.28–5.95), sugar depletion, and organic acid accumulation depending on the microbial strain employed, indicating sensitivity to acidic and osmotic conditions during bioprocessing [16]. Additionally, metabolomic evaluations in amaranth cultivated under rhizospheric modulation revealed significant shifts in energy metabolism pathways, as evidenced by multivariate analyses explaining up to 75.06% of total variance, suggesting that native associated microorganisms and substrates are highly responsive to environmental perturbations [94]. Bioencapsulation processes face similar challenges, as pressure conditions, homogenization, and spray-drying can damage microparticles or alter the release of bioactive compounds, thereby reducing their functionality.

Moreover, the costs associated with scaling—related to specialized equipment, online sensors, regulatory validation, and energy required to maintain optimal conditions—remain high, limiting the adoption of these technologies in regions where Andean crops are predominant but industrial infrastructure is still insufficient. Likewise, the lack of harmonized standards for ingredients derived from advanced bioprocesses represents a barrier to their certification as functional foods, particularly when novel metabolites, such as oxindole acetates identified through high-resolution platforms, are incorporated [98]. These limitations highlight the need to optimize quality control of Andean matrices, reduce analytical equipment costs, and scale fermentation and encapsulation technologies toward modular and energy-efficient models. Such improvements will help bridge the gap between omics research and sustainable industrial production.

### 7.3. Analytical Limitations and Gaps in Metabolomic and Proteomic Characterization

**Figure 6:**
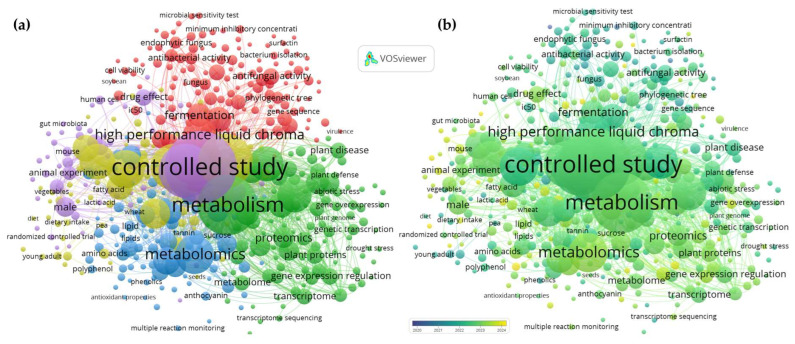
Network visualization of keyword co-occurrence generated with VOSviewer (overlay visualization) for advances in proteomics and metabolomics in Andean grains and tubers. (**a**) Network structure of keyword co-occurrence; (**b**) Overlay visualization showing temporal or density-based color gradients.

The metabolomic and proteomic characterization of Andean grains and tubers continues to face methodological limitations that constrain result interpretation and the standardization of functional biomarkers. Although techniques such as LC-MS/MS, UHPLC, GC-MS, and high-resolution mass spectrometry have enabled the identification of complex profiles—including bioactive peptides in quinoa, betalains in amaranth, and differentiated anthocyanins in purple-fleshed potatoes—there remains a lack of databases specific to Andean species (Figure 6). This absence complicates automatic annotation, increasing the proportion of “unidentified” compounds and limiting reproducibility across laboratories. Despite significant advances in the analysis of Andean grains and tubers, methodological constraints still restrict the comprehensive characterization of their metabolites and proteins. Even though recent studies have employed high-resolution platforms such as LC-MS/MS, UHPLC-QTOF, Orbitrap, and multiomic approaches, the chemical diversity of these matrices surpasses the detection and structural annotation capabilities of current methods. For instance, studies in sweet potato identified over 4447 secondary metabolites [95], and those in quinoa identified up to 1060 metabolites and 13,095 differentially expressed genes [68]; however, a considerable proportion remains unannotated due to the lack of species-specific libraries.

Furthermore, the intrinsic variability of native matrices, influenced by altitude, climate, water stress, and agricultural practices, generates fluctuations that demand strict sampling and normalization protocols. Added to this are the challenges of quantifying thermosensitive or low-concentration metabolites, which are often lost during prior processing or masked by matrix interferences. In proteomics, advances are also not without gaps: the quantification of apoptotic biomarkers in quinoa [64] and the detection of 693 differential proteins in potato leaves using iTRAQ [71] highlight the complexity of the plant proteome but also reveal limitations in mapping isoforms, post-translational modifications, and low-abundance peptides. Similarly, peptide profiles with bioactive activity, such as the 91 peptides identified after quinoa protein fermentation [22] or the CEase and PL inhibitory peptides [41], require deeper validation integrating quantitative mass spectrometry, molecular docking, and standardized biofunctional assays.

Integrated metabolomics and proteomics are also not applied systematically, which limits the ability to correlate key metabolites with biochemical pathways of industrial interest. Overcoming these gaps requires the development of species-specific molecular libraries, pretreatment methods adapted to Andean matrices, and multi-omics strategies that enable a higher-resolution understanding of the inherent functionality of these biological resources. The absence of analytical protocols hampers cross-study comparability, as reflected in the variability of flavonoid reports, with 154 compounds identified in quinoa [69] and 90 flavonoids in integrated studies [77]. Likewise, metabolomic analyses depend heavily on instrumental parameters—such as column size, *m*/*z* range, or acquisition speed—which generate substantial differences even within the same species, as observed in potato leaves analyzed with high-speed LC-MS at 4 scans/s [66]. In this context, a significant gap remains in the integration of multi-omics data; although some studies combine transcriptomics and metabolomics [68,75,76], the lack of standardized bioinformatic models limits the functional interpretation of Andean metabolism under stress, processing, or fermentation conditions.

### 7.4. Regulatory Barriers and Standardization of Functional Ingredients

**Figure 7:**
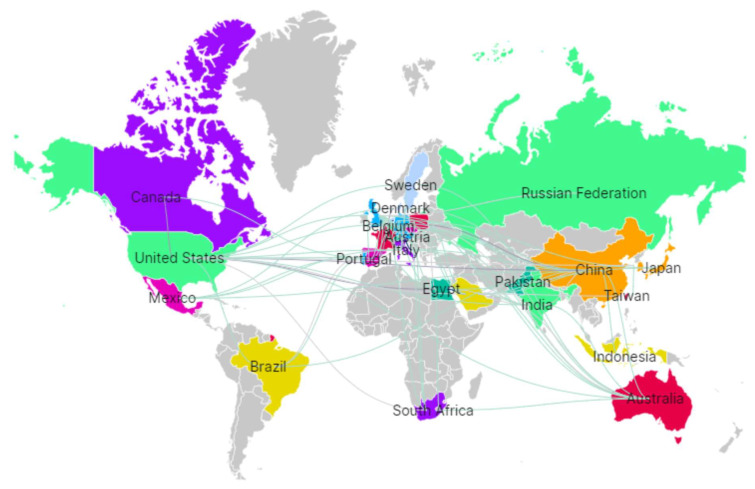
Co-Authorship Countries in Emerging Topics on Food Transformation.

The regulation of functional ingredients derived from Andean grains and tubers remains one of the main obstacles to their full entry into international markets. Although numerous bioactive compounds—such as anthocyanins from purple potato, betalains from amaranth, or functional peptides obtained through fermentation—have demonstrated beneficial properties, their commercial approval requires robust evidence regarding safety, stability, and physiological efficacy. Regulatory frameworks in the European Union and the United States typically demand toxicological assessments, allergenicity analyses, genetic traceability, and proof of effects in in vivo models or preliminary clinical trials (Figure 7). According to bibliometric analyses of keyword co-occurrence and collaborative countries, research activity is predominantly concentrated in Canada, China, and the Russian Federation. These regulatory requirements are particularly challenging for native matrices whose geographic and seasonal variability makes it difficult to present uniform chemical profiles.

Regulatory barriers and the lack of standardization remain critical challenges for the commercialization of functional ingredients derived from Andean crops. Advanced metabolomic studies have revealed an extraordinary chemical complexity, such as the identification of 4447 secondary metabolites in sweet potato varieties using UHPLC-MS [41] and the discovery of fourteen novel oxindoleacetates in quinoa through high-resolution platforms (UHPLC-QTOF/Orbitrap) [106]. While these findings highlight strong functional potential, they also complicate regulatory approval due to difficulties in defining consistent chemical markers and specifications. Similarly, targeted metabolomics in amaranth inoculated with Glomus spp. demonstrated clear metabolic shifts affecting energy metabolism pathways [16], underscoring how agronomic and biological variables influence ingredient composition.

Functional coatings and indicator films developed from potato and sweet potato matrices have shown promising quality and safety improvements [99,105], yet their acceptance requires harmonized protocols for efficacy, safety, and stability assessment. Moreover, fermentation-driven enhancements in sweet potato bioactive compounds [19] further emphasize the need for standardized processing and analytical frameworks to ensure reproducibility, regulatory compliance, and international market acceptance of Andean functional ingredients. In this context, the global co-authorship network illustrated in Figure 7 reinforces these challenges and opportunities, revealing strong collaborative links among Canada, China, the Russian Federation, Europe, and Asia–Pacific regions. Such international interactions support methodological harmonization, regulatory dialogue, and knowledge transfer, which are critical to overcoming standardization barriers and facilitating the global commercialization of functional ingredients derived from Andean matrices.

The lack of international standards for quantifying metabolites and functional parameters represents another significant barrier. While some countries accept methods based on LC-MS/MS or FTIR spectroscopy to certify bioactive compounds, others require interlaboratory validation and pharmacopoeia-based norms that are not well suited to complex plant matrices. This disparity affects the comparability of studies and hinders the development of the technical documentation required for product registration. The consolidation of functional ingredients derived from Andean grains and tubers faces substantial regulatory barriers in Latin America, Europe, and Asia, particularly regarding the classification, validation, and authorization of bioactive compounds obtained through controlled fermentation, bioencapsulation, and advanced omics technologies. Despite the experimental evidence supporting their bioactivity, such as the high polyphenol content in encapsulated native potato reaching up to 9.86 mg GAE/g [40], the 14 newly identified oxindole acetate conjugates in quinoa [106], or the CEase and PL inhibitory peptides with antihypercholesterolemic potential [41], current regulatory frameworks do not fully account for the complexity of these products.

### 7.5. Geographical Concentration of Research and Limitations for Global Extrapolation

A major limitation of current research on the fermentation and bioencapsulation of Andean grains and tubers is its predominant concentration on native South American matrices, particularly quinoa, amaranth, potato, sweet potato, mashua, ulluco, and arracacha, which are endemic to the Andean region and traditionally processed within Latin American food systems. Most functional evidence—including the enhancement of phenolic content, antioxidant activity, and protein digestibility through fermentation, as well as the release of bioactive peptides with antihypertensive or hypoglycemic potential—has been generated using regional cultivars and locally sourced substrates [29,34,92]. Likewise, metabolomic and peptidomic studies identifying oxindoleacetate conjugates, cultivar-dependent phenolic variability, and low-molecular-weight bioactive peptides in quinoa, potato, and sweet potato rely almost exclusively on Andean germplasm [65,95,98]. Encapsulation technologies employing native starches, alginate, pectin, and modified Andean biopolymers—such as nanoencapsulation of potato phenolics, alginate-based peptide beads, and bioencapsulation of nodal segments for genetic conservation—have similarly been optimized under region-specific conditions [40,45]. While this geographical focus is essential for valorizing Andean biodiversity and preserving traditional knowledge, it constrains the global extrapolation of functional performance, fermentation kinetics, and encapsulation efficiency, as these parameters are highly sensitive to cultivar genetics, environmental conditions, and postharvest practices. Addressing this regional concentration through comparative, multi-environmental studies is therefore critical for the international positioning of Andean-derived nutraceutical foods.

### 7.6. Innovative Perspectives for the Development of Functional Foods Based on Biotransformation

Biotransformation has become a strategic platform for designing next-generation functional foods, driven by advances in fermentation, germination, and specialized enzymatic pathways. Future perspectives for the development of functional foods derived from Andean matrices rely on recent progress in biotransformation, fermentation, encapsulation, and biomaterial design. Fermentative bioprocesses have shown particular promise, as demonstrated in sweet potato, where fermentation with *A. niger* and *B. coagulans* significantly increased the content of phenols, amino acids, and organic acids, while also improving sensory properties, highlighting its potential as a platform for microbially based functional foods [16]. The generation of bioactive peptides through ultrasonic hydrolysis of sweet potato and enzymatic treatments in quinoa has enabled the production of antioxidant fractions with inhibitory activity against key enzymes associated with hypercholesterolemia, creating opportunities for the development of plant-based nutraceuticals [41,99].

One of the most relevant future perspectives is the application of targeted microorganisms and engineered microbial consortia capable of modulating specific metabolomic profiles, promoting the formation of bioactive compounds with antioxidant, anti-inflammatory, prebiotic, or immune-modulating functions. This approach helps overcome the inherent variability of traditional processes, enabling the production of stable, reproducible matrices with more robust functional profiles. In the same direction, an emerging focus in this field is nanoencapsulation and microencapsulation, which are valuable for stabilizing phenolic compounds and improving their bioavailability. In native potato, optimized nanocapsules have yielded stable particles of 133–165 nm with controlled release, strengthening the design of robust functional ingredients for complex matrices [40]. Complementarily, protein–pectin coacervates in amaranth have shown the ability to incorporate phenolic extracts and enhance antioxidant activity, suggesting strong potential for application in clean-label formulations [100]. Postharvest biotechnology also contributes important innovations, including edible coatings and bioactive films derived from amaranth or tubers, which can improve quality, stability, and functionality in fresh and minimally processed products [46,55,96,97].

Likewise, another key trend reflected in the evolution of food transformation is the use of smart bioreactors, solid-state fermentations, and synergistic systems that combine bacteria, yeasts, and fungi to enhance the release and conversion of essential nutrients. These technologies promote sustainability, reduce the need for additives, and enable the production of foods with greater functional stability. Advances in metabolomics and the discovery of new bioactive compounds continue to reinforce the functional value of Andean crops. The identification of previously unreported oxindole acetates in quinoa using UHPLC-QTOF/QOrbitrap indicates the presence of metabolic routes that remain underexplored and that hold significant functional potential [98]. Similarly, metabolomic profiles of sweet potato and amaranth reveal remarkable diversity in nutrient-relevant, antioxidant, and bioactive metabolites, supporting their use in the design of next-generation functional foods [65,93,94,95].

### 7.7. Integration of Emerging Technologies for Future Applications in Andean Grains and Tubers

The integration of emerging technologies represents a decisive opportunity to enhance the functional value of Andean grains and tubers through more precise, sustainable, and industry-aligned processes. One of the most promising avenues is the implementation of sensor-assisted fermentation, real-time analytics, and digital control systems, which enable dynamic adjustments of parameters such as pH, oxygenation, and enzymatic activity, thereby optimizing the generation of bioactive compounds. For instance, a study on ultrasonic hydrolysis applied to sweet potato demonstrated the production of low-molecular-weight antioxidant peptides, highlighting its applicability in hybrid processes that integrate acoustic cavitation with enzymatic action [99]. Emerging technologies thus offer a strategic pathway to strengthen the biological and functional value of Andean crops, supporting the transition toward more efficient, sustainable, and health-oriented food systems. The combined use of biocatalysis, fermentation, and physical technologies shows strong potential for producing next-generation bioactive ingredients. Similarly, the production of bioactive peptides in quinoa through enzymatic digestion and molecular analysis underscores the relevance of integrating biocatalysis with computational modeling to accelerate the discovery of functional compounds [41].

On the other hand, the combined use of omics tools, computational modeling, and machine-learning platforms is enabling more accurate predictions of how Andean matrices respond to different biotransformation schemes. This approach facilitates the design of targeted metabolomic profiles, the selection of microorganisms with specific functional roles, and the accelerated evaluation of new formulations without the need for prolonged experimental testing. These innovations are further complemented by advances in smart bioencapsulation, nanotechnology, and hybrid matrices based on polysaccharides or plant proteins, which can protect sensitive metabolites and enhance their release within the body. Advanced metabolomic tools, including UHPLC-QTOF, UHPLC-QOrbitrap, and LC-MS, have become essential technologies for the in-depth characterization of metabolites in Andean crops. Their application has led to the identification of previously unreported compounds, such as novel oxindole acetates in quinoa [98], and the mapping of thousands of differential metabolites in sweet potato and amaranth, providing critical information to guide process optimization, functional food design, and the selection of varieties with superior bioactive profiles [65,92,93,94,95].

## 8. Conclusions

Microbial fermentation and bioencapsulation of Andean grains and tubers represent innovative tools to enhance their nutritional, functional, and sensory value. The studies reviewed indicate that fermentative processes using strains such as *Aspergillus niger* and *Bacillus coagulans* significantly increase protein content, free amino acids, and phenolic compounds in matrices such as sweet potato and quinoa, improving digestibility and flavor profiles. Bioencapsulation strategies—including nanocapsules, edible coatings, and nodal-segment encapsulation—ensure the protection, stability, and controlled release of bioactive compounds such as polyphenols, flavonoids, and antihypercholesterolemic peptides during storage, processing, and gastrointestinal digestion.

In this context, sustainable nutraceutical foods can be defined as food systems derived from natural matrices that deliver scientifically validated bioactive compounds at effective levels, supported by advanced processing technologies, while simultaneously preserving agrobiodiversity, reducing processing losses, and enabling environmentally responsible production chains. Unlike conventional functional foods, which are primarily characterized by general health-promoting effects linked to nutrient fortification or fiber enrichment, nutraceutical foods exhibit targeted physiological activities, such as antioxidant, antihypertensive, or lipid-regulating effects, supported by proteomic, metabolomic, and bioavailability evidence.

Advanced omics analyses have identified novel functional metabolites, including oxindole acetate conjugates and peptides with antioxidant and lipase- or cholesterol-esterase-inhibitory activity, reinforcing the nutraceutical potential of Andean crops. Furthermore, the integration of bioencapsulation with micropropagation and germplasm conservation strategies contributes to sustainability by ensuring high-quality plant material and long-term preservation of native genetic resources. Overall, the convergence of controlled fermentation, bioencapsulation, and omics-based validation supports the development of sustainable Andean nutraceutical foods, positioning traditional grains and tubers as strategic resources for health-oriented, environmentally responsible food systems.

## Figures and Tables

**Table 1:** Search strategies used in databases.

Database	Strategy	Search Variables
Web of Science	Encapsulation and Andean Matrices	(“Bioencapsulation”) AND (“Andean grains)
Scopus	Proteomics, Functionality, and Fermentation Systems Applied to Andean Matrices	(“Proteomics”) AND (“Fermentation”) AND (“Andean grains”)

**Table 2:** Types of Fermentation Used in Functional Matrices.

Matrix or Species	Type of Fermentation/Microorganism	Results	Ref.
Amaranth and quinoa (flours)	Liquid sourdough fermentation with *Weissella cibaria* C43-11 and *Lactobacillus plantarum* ITM21B for 15 h.	High exopolysaccharide production (~20.79 g/kg at 250 DY); significant protein degradation (~51%); increase in organic acids and improved sourdough texture.	[15]
Sweet potato (slurry)	Submerged fermentation with various strains: *B. coagulans*, *S. cerevisiae*, *L. plantarum*, *B. subtilis*, *B. breve*, *L. acidophilus.*	49 volatile compounds produced; *B. coagulans* yielded the richest ester profile and highest total acidity; aromatic profile shifted toward fermentative alcohols and aldehydes.	[16]
Grain mixture: wheat, oats, brown rice, barley, quinoa, lentils	Solid-state fermentation (SSF) with *Bacillus amyloliquefaciens* 245.	Increased essential amino acids, higher amylase and protease activity, increased phenolic compounds and antioxidant capacity after 36 h.	[21]
Quinoa (proteins)	Fermentation to obtain bioactive peptides with *Lactobacillus paracasei* CICC 20241.	91 peptides were identified, including medium-chain peptides with ACE-inhibitory activity (IC_50_: 40–80 μM).	[22]
Sweet potato (protein gel)	Controlled fermentation prior to protein gel formation.	Improved color stability and anthocyanin retention against oxidation; efficiency increased to 87.27%.	[26]
Amaranth (proteins)	Fermentation to release enzyme-inhibitory peptides—*Lactobacillus* spp.	Increased peptides with inhibitory capacity IC_50_ = 0.47 mg/mL; peptide profiles associated with antioxidant and antihypertensive pathways.	[27]
Sweet potato, soybean, and agro-industrial residues	Fermentation with medicinal fungus *Ganoderma lucidum.*	Increased antioxidant compounds and bioactive polysaccharides in substrates: sweet potato and soybean residues at 11.43, 32.64, 40.19 μmol Trolox/100 g and 19.29, 17.70, 32.35 μmol Trolox/100 g, respectively.	[28]
Buckwheat + quinoa	Germination followed by co-fermentation (controlled co-fermentation).	Improved phenolic profile, increased antioxidants, and reduced antinutrients; tannin reduction: buckwheat 83%, quinoa 20%.	[29]
Moromi and shochu	Fermentation with temperature variation (technological reference).	Regulation of lactic acid kinetics and enzymatic activity; amino acids higher in moromi fermented at 38 °C, while shochu at 25 °C exhibited more fruity notes.	[32]
Saponins + fungi − fermentation optimization	Fermentation to degrade saponins with specialized fungi.	Reduced bitterness; kinetic parameters optimized secondary metabolites; potato concentration 97.3 mg/mL, glucose 20.6 mg/mL, pH 2.1 at 29.2 °C for 6 days.	[33]
Ulluco and arracacha	Fermentation as a process to intensify bioactive compounds.	Increased phenolic content and antioxidant capacity; transformation of structural carbohydrates; fiber content: ulluco 14.00%, xanthorrhiza 4.07%.	[34]

Note. The details presented in the table relate to the matrix or species, type of fermentation/microorganism, and the results obtained in experimental studies conducted between 2020 and 2025. Abbreviations: C43, *Weissella cibaria strain*; ITM21B, *Lactobacillus plantarum strain*; DY, Dough Yield; *B. coagulans*, *Bacillus coagulans*; *S. cerevisiae*, *Saccharomyces cerevisiae*; *L. plantarum*, *Lactobacillus plantarum*; *B. subtilis*, *Bacillus subtilis*; *B. breve*, *Bifidobacterium breve*; *L. acidophilus*, *Lactobacillus acidophilus*; SSF, solid-state fermentation; CICC 20241, *Lactobacillus paracasei strain*; ACE, angiotensin-converting enzyme; IC_50_, compound concentration.

**Table 3:** Bioencapsulation and Nanoencapsulation Technology in Andean Matrices.

Matrix/System	Technology/Encapsulating Material	Results	Ref.
Carrot (phenolic extract) + Potato (protein matrix)	Potato protein–pectin coacervate; emulsification.	Average particle size ranged from 65.05–152.47 µm. Encapsulation efficiency (EE) increased (69.26–90.15%), while apparent retention (AR) decreased (53.90–102.16%) upon emulsification.	[36]
Starch hydrogels (model application)	High-pressure-processed (HPP) starch hydrogels.	HPP starch hydrogels (600 MPa, 15 min) showed stable gelatinization. The extract affected color and released polyphenols in a controlled manner, confirmed by FT-IR and Franz diffusion.	[37]
Amaranth hydrolysates (peptides)	Alginate–pectin beads (ionic gel)	AG–PC beads showed increasing sizes with higher PC content. Encapsulation reached 95.57%. ACE-inhibitory activity reached 97.97% and was maintained after in vitro digestion.	[9]
Carrot (protein matrix: soy/potato)	Protein matrices + starch; film/spray-drying encapsulation.	Beads showed EE of 70.93–82.59% and sizes of 2.18–2.64 mm; 50% IPS–50% starch blends provided superior carotenoid protection, extending shelf life up to 106 days.	[38]
Potato (peptidic)—digestion/stability	Protein matrices and release systems.	Structures showed DPPH activity of 3–20%. Heated gels exhibited notable antioxidant capacity.	[50]
Quinoa (proteins)—emulsion gels	Quinoa protein-based emulsion gels.	QPH increased S_0_ (*p* = 0.006) and emulsifying activity (*p* = 0.002), but reduced stability (*p* < 0.000). Water-holding capacity ≈ 70%. Hardness decreased (*p* < 0.000). Concentrations 0.5–2.0% formed well-defined 3D networks.	[48]
Sweet potato compounds (extracts)	Beads/alginate/ionic gelation; active films.	Encapsulation reached 60% carotenoids and 61–64% phenolics. Retention after 60 days: 43–59% under light/dark conditions. Degradation kinetics: k = 0.0149–0.0106 d^−1^.	[49]
Ferulic acid (antioxidant) in quinoa (nanoformulation)	Quinoa protein + zein nanoparticles (antisolvent precipitation).	Quinoa prolamin showed higher EE (81.2%) and loading capacity (LC, 29.7%). Zein reached 70.7% EE and 18.5% LC. Enhanced gastric resistance and intestinal release of ferulic acid.	[51]
Anthocyanins (purple potato)	Spray-drying microencapsulation (quinoa starch/gum arabic).	Encapsulation achieved 86% EE, reduced degradation, and increased anthocyanin bioaccessibility by 20%, maintaining stability during storage and digestion.	[17]
Anthocyanins (maize + potato)	Microencapsulation in mixed matrices (starch/gum).	*S. tuberosum* CI_50_ = 0.070 mg/mL; minimum viability. *Z. mays* CI_50_ = 0.275 mg/mL; gradual reduction, slopes −41.83 vs. −7.32, respectively.	[52]
Extract (quinoa starch + Tara gum)	Quinoa starch + Tara encapsulation (spray/coacervation).	Capachu microcapsules contained 9.60 mg GAE/g phenolics, 211.40 mg C3G/g anthocyanins, 142.43 µmol/g DPPH, releasing 24.04 mg GAE/g.	[53]
Mashua (extracts)	Microencapsulation with modified Andean starches (OSA).	Optimized mashua extract (160 °C, 2% OSA) achieved higher EE, phenolics, and antioxidants, with low a_w_ and hygroscopicity using pink oca OSA.	[39]
Native potato phenolics	Nanoencapsulation (spray-drying/nanoprecipitation)	Optimal encapsulation (120 °C, 141 L/h) yielded high EE, elevated DPPH, particle size 133–165 nm, negative ζ, low aw/moisture, and maximum release 9.86 mg GAE/g.	[40]
Quinoa bioactive peptides (applications)	Peptide formulation for stability and release (micro/nano).	Quinoa hydrolysates with chymotrypsin showed strong inhibition: CEase CI_50_ = 0.51 mg/mL; PL CI_50_ = 0.78 mg/mL; 4–12 active peptides identified.	[41]
Plant matrices with yeast (microencapsulation)	Microencapsulation using yeast particles + polysaccharide matrices.	YGP water showed 37.8% lower RR; acid/alkaline hydrolysis increased ARR 14.8–27.8%; organic solvents released more anthocyanins than control.	[54]
Edible coatings on fresh potato	Alginate + essential oils/chitosan.	Formulations F1–F2 increased chroma; F1–F4 elevated anthocyanins after 3 months (*p* < 0.05); alginate improved color, gloss, and sensory acceptability.	[55]
Composite films with microcapsules (potato)	Film-forming with microcapsules (alginate/biopolymer).	Microcapsules achieved 87% EE; film with 4.5% LM showed 46% antioxidant activity, improved stability, blocked UV, and extended blueberry freshness.	[42]
Jasmonate microcapsules (agro)	Microcapsules for post-harvest regulation.	MeJA 300 μmol/L was optimal; microcapsules with 2.5% alginate + 0.5% chitosan enhanced preservation using lower dose than solution.	[43]
Functional oil encapsulated in hydrocolloids	Oil-in-hydrocolloid microcapsules for product reformulation.	Encapsulated oil increased thermal diffusivity and reduced baking time, boosting productivity 17%; gluten-free cookies showed higher porosity and improved lipid profile.	[44]
Potato—response to encapsulating agents in culture	Encapsulation agents in culture stations.	Encapsulation with 4% alginate, 100 mM CaCl_2_ and ½ MS achieved 99% conversion, 17% abscission, and rapid shoot development, optimizing micropropagation.	[45]
Active films based on amaranth	Active films (amaranth protein/hydrolysates + additives).	Glycerol increased EB to 12.19% and reduced TS to 1.12 MPa; 2% phenolics decreased EB and TS due to agglomeration.	[46]
Nanoencapsulation of potato compounds (various formulations)	Nano-/microencapsulation (spray/nanoemulsion).	At 116 °C and 15% encapsulant, higher phenolic retention, improved antioxidant capacity, and lower moisture and aw were achieved, outperforming 96°C–25% conditions.	[47]
Industrial coatings and related technologies	Edible coatings and drying processes.	Coatings improved weight loss and firmness in RG and PM at 55 ± 5% RH and 5 ± 1 °C; inhibited sprouting under storage.	[56]
Fresh cut purple sweet potato.	Surface bioencapsulation with chitosan (Ch) and sodium alginate (SA) composite gel.	ChCSA coating reduced color change to ΔE = 8.97, versus Ch (16.86), SA + C (13.05) and control (22.90), forming a barrier limiting light and oxygen, preserving color for 12–18 days.	[57]
Gluten hydrolysate obtained with pancreatin	Spray-drying using maltodextrin, potato starch, and blends (30:70).	Higher water activity (a_w_ = 0.36), high encapsulation efficiency (85.79%), moisture 8.20%; increased solubility and density with more maltodextrin; spherical and rough microcapsules; improved antioxidant stability and controlled release under simulated gastrointestinal digestion.	[58]

Note. The details presented in the table are related to the matrix or species, the encapsulation technology/material, and the results obtained in experimental studies conducted during the period 2020–2025. Abbreviations: EE, encapsulation efficiency; RA, anthocyanin retention; HPP, high-pressure processing; FT-IR, Fourier-transform infrared spectroscopy; AG-PC, alginate–pectin biopolymers; ACE, angiotensin-converting enzyme; IPS, soy protein isolate; DPPH, 2,2-diphenyl-1-picrylhydrazyl radical; QPH, quinoa protein hydrolysates; So, surface hydrophobicity; WHC, water-holding capacity; LC, loading capacity; AF, ferulic acid; MD, maltodextrin; IC50, compound concentration; GAE, gallic acid equivalents; C3G, cyanidin-3-glucoside; OSA, modified Andean starches; PL, pancreatic lipase; CEase, cholesterol esterase; YGP, yeast glucan; ARR, red radish anthocyanins; F1, sodium alginate; F2, sodium alginate and potato starch; F4, chitosan, sodium alginate and potato starch; LM, lycopene microcapsules; UV, ultraviolet light; MeJA, methyl jasmonate; MS, Murashige and Skoog; EB, elongation at break; TS, tensile strength; RG, Rio Grande Russet; PM, Purple Majesty; HR, relative humidity; ChCSA, gel coating; Ch, chitosan; SA + C, sodium alginate + calcium chloride.

**Table 4:** Proteomics, Metabolomics, and Omics Approaches Applied to Andean Matrices.

Matrix or Species	Proteomic and Omics Technique	Results	Ref.
Amaranth (seeds)	LC-MS (betalains).	LC-MS identified 30 betacyanins and 13 betaxanthins in *A. cruentus* with <5 ppm accuracy, confirming its value as a pigment source.	[62]
Potato (white vs. purple flesh)	Metabolomics + Transcriptomics (UHPLC-MS/MS + RNAseq).	UPLC-MS/MS identified 18 anthocyanins associated with 12 genes; *St5GT* showed strong overexpression in purple potato, confirming its key pigment role.	[63]
Quinoa (grains)	Comparative proteomics LC-MS/MS.	LC-MS/MS quantified 13 apoptotic biomarkers; 12 responded to quinoa proteins, showing increases similar to paclitaxel after 72 h.	[64]
Sweet potato (Ipomoea batatas)	Metabolomics (LC-MS).	LC-MS/MS identified 527 amino acids, 556 organic acids, and 39 lipids; CS showed more essential amino acids, ZS notable for succinic acid.	[65]
Potato leaves (field-grown)	Comparative proteomics.	LC-MS analyzed samples using 2 µL injection, 2.1 × 50 mm column, flow 0.5–0.8 mL/min, *m*/*z* range 70–1700, acquisition 4 scans/s.	[66]
Quinoa (seeds)	Saponin profiling (metabolomics).	114 accessions evaluated; saponins ranged 0.22–15.04 mg/g; 75% were low; 12 oleanane saponins and one novel compound were identified.	[67]
Quinoa (seeds/seedlings)	Integrated metabolomics + transcriptomics.	1060 metabolites and 13,095 differential genes detected; lipids and flavonoids predominated, highlighting hormonal signaling and AP2/ERF in response to high humidity.	[68]
Quinoa (cultivars)	Metabolomics (targeted/untargeted).	154 flavonoids and 39,738 genes identified; 11 metabolites and 22 genes explained biosynthetic variation across four developmental stages.	[69]
Quinoa (extracts)	Peptidomic analysis.	pH 2 fraction showed 2451 U/mL protease and strong antibacterial, antifungal, and anticancer activity against A549 and HeLa cells.	[70]
Potato leaves (toxin response)	Quantitative iTRAQ proteomics.	693 differential proteins identified: 460 upregulated, 233 downregulated, highlighting changes in metabolic pathways and plant defense against taxtomin A.	[71]
Potato (Bulgarian cultivars)	Metabolomics (profiling).	Total phenolics ranged: flesh 318–636 mg/100 g, skin 2847–4120 mg/100 g; flavonoids: flesh 1.21–2.26 mg/100 g, skin 32.50–84.40 mg/100 g.	[72]
Quinoa (colored varieties)	Non-targeted metabolomics (LC-MS).	689 compounds identified; 251, 182, and 317 varied among groups. Notable: 22 flavonoids, 5 phenolic acids, and 1 differential betacyanin.	[73]
Quinoa (phenolics)	Spectrometry and chromatography (HPLC-MS)	430 polyphenols identified: 121, 116, and 148 differentials. Black quinoa had 643.68 mg/100 g phenolics; white quinoa IC50: 3.97 and 1.08 mg/mL.	[74]
Quinoa (colors/cultivars)	Metabolome + transcriptome.	Four cultivars showed distinct profiles; 6 enriched pathways; multiple amino acids, tannins, lipids, and alkaloids varied significantly among analyzed grains.	[75]
Quinoa (seeds/seedlings)	Transcriptomics and metabolomics (low-temperature stress).	Two quinoa variants evaluated at −2, 5, and 22 °C; 794 metabolites and 52,845 genes detected, including 6628 novels.	[76]
Quinoa (flavonoids)	Integrated omics (transcriptomics + metabolomics).	Black, red, yellow, and white quinoa seeds analyzed; 90 flavonoids detected, 18 key metabolites, 25 regulatory genes identified.	[77]
Triticale/comparative cereal	Metabolomics (harvest/temporal changes).	*Triticosecale* ‘Bilinda’ showed 93 polyphenolic compounds, including 9 flavones, 7 flavonols, 2 flavan-3-ols, 5 hydroxybenzoic acids, and 4 carotenoids.	[78]
Potato (Snakin2 study)	Proteomics/functional (biochemistry).	RNAi7: COMT and CAD 5–10×, Prx10 maximum; OE27: minor changes; peroxidase, COMT, CAD activities ↑; StSN2 interacts with Prx2, Prx9, Prx10.	[61]
Potato hybrids (field)	Integrated proteomics + transcriptomics.	High α-solasonine and α-solamargine; SBT1.7 X2 and subtilisin protease ↑; carbonic anhydrase and miraculin ↓; endo-1,3-β-glucanase 47.96× higher.	[79]
Amaranth (polyphenols/flavonoids)	Metabolomics (profiling).	LS7 showed maxima: provitamin A 2–3×, high vitamin C, total phenolics and flavonoids elevated; antioxidant capacity (DPPH/ABTS) higher; LS9 slightly lower.	[80]
Cereals and Andean grains (oats, barley, quinoa QMM and QKU)	LC-MS/MS (mycotoxin metabolomics).	European cereals contained Fusarium mycotoxins —HT2, MON, NIV—in >50% samples, with INFE and non-specific metabolites (CTO, CDP-Tyr, CDP-Val, EMO, ENC). Andean grains: quinoa QMM showed low toxic load except some Fusarium and Alternaria mycotoxins; QKU had high non-specific metabolites AsG (89), AsP (90), and NBP (97).	[81]
Potato (bacterial isolates Priestia megaterium)	Oxford Nanopore genomic sequencing, bioinformatic annotation, and optimized MS/MS.	AuNPs increased metabolites of 254–270 Da depending on concentration, confirmed by genomic data and biosynthetic profiles of the isolate.	[82]

Note. The details presented in the table are related to the matrix or species, proteomic and omics technique, and the results obtained from experimental studies conducted between 2020 and 2025. ↑ indicates an increase, ↓ indicates a decrease. Abbreviations: LC, liquid chromatography; MS, mass spectrometry; UPLC-MS/MS, ultra-performance liquid chromatography–electrospray ionization–mass spectrometry; RNAseq, ribonucleic acid sequencing; St5GT, Solanum tuberosum glucosyltransferase 5; LC-MS/MS, liquid chromatography–mass spectrometry; CS, orange sweet potato flesh; ZS, purple sweet potato flesh; AP2/ERF, Apetala2/Ethylene-Responsive Factor; HeLa, cell lines; iTRAQ, Isobaric Tags for Relative and Absolute Quantitation; IC50, half-maximal inhibitory concentration; RNAi7, Ribonucleic Acid interference 7; COMT, Caffeic Acid O-Methyltransferase; CAD, Cinnamyl Alcohol Dehydrogenase; Prx10, peroxidase 10; OE27, Over-Expression 27; StSN2, *Solanum tuberosum* Snakin-2; Prx2, peroxidase 2; Prx9, peroxidase 9; SBT1.7X2, Subtilisin-like protease transcript variant X2; LS7, Line Selection 7; TP, Total Phenolics; TF, Total Flavonoids; AC, Antioxidant Capacity; DPPH/ABTS, DPPH radical (2,2-diphenyl-1-picrylhydrazyl)/ABTS^+^ radical cation (2,2′-azino-bis(3-ethylbenzothiazoline-6-sulfonic acid)); HT2, Hotelling’s T-squared; MON, Moniliformin; NIV, Nivalenol; CTO, unspecific metabolites.

**Table 5:** Functionality, Bioactive Compounds, and Applications of Andean Matrices.

Matrix or Species	Method or Approach	Results	Ref.
Quinoa (red)	Enzymatic assays (α-glucosidase inhibition), phenolic analysis (HPLC).	Red quinoa BPE IC_50_ α-glucosidase 10.295 mg/mL, higher antioxidant activity (DPPH/ABTS), delayed starch digestion, reduced postprandial glucose at 50 mg/kg.	[92]
Amaranth (grain/leaves)	Metabolomic profiling (LC-MS), bioactive compound analysis.	Caffeic and glucaric acids increased 2.9–5.2% after cooking; domestic oxidation reduced phenolics 22–60%; buns decreased TPC by up to 60%.	[93]
Amaranth inoculated with Glomus (rhizosphere)	Targeted metabolomics.	PCA: PC1 explained 49.65%; PC1 + PC2 75.06%; OPLS-DA clearly discriminated control vs. treated; metabolites mainly affected energy metabolism pathways.	[94]
Sweet potato (varieties)	Untargeted metabolomics (UHPLC-MS)	4447 secondary metabolites identified; CS vs. BS: 1540, ZS vs. BS: 1949, ZS vs. CS: 1931; 20 flavonoids and 13 common phenolic acids.	[95]
Potato (functional coatings)	Evaluation of edible coatings + quality assays (firmness, color, microbial).	AEC-TEO 0.05% increased L to 10.55, firmness to 8.24 N, reduced browning 4.19, decreased microbes 1.21–3.63 log CFU/g.	[96]
Sweet potato (indicator films)	Development of indicator films (sensory/colorimetric).	Optimal temperature 30 °C: elongation 98.46%, tensile strength 95.73, ΔE and permeability decreased, excellent pH and NH_3_ sensitivity, lighter color.	[97]
Quinoa (phytohormones/auxins)	Chemical-metabolomic analysis of auxins and derivatives.	14 new oxindoleacetates identified in quinoa via UHPLC-QTOF-MS/MS and UHPLC-QOrbitrap-MS/MS, present in conventional and organic crops.	[98]
Sweet potato (post-fermentation)	Fermentation studies and functional measurements (antioxidants, volatiles).	Fermented sweet potato: pH 3.28–5.95; sugars ↓; protein ↑; phenolics ↑ (*A. niger*); amino acids + 64.83% (*B. coagulans*); lactic acid ↑; improved flavor.	[16]
Sweet potato (varieties by color)	Comparative nutrient and metabolomic profiling.	Metabolomic analysis of sweet potatoes: 527 amino acids, 556 organic acids, 39 lipids; CS had higher essential amino acids; ZS characterized by succinic acid.	[65]
Potato (encapsulated phenolics)	Micro/nanoencapsulation and functional activity assays.	Optimization of nanoencapsulation of native potato phenolic extracts: 120 °C, 141 L/h; nanocapsules 133–165 nm, maximum release 9.86 mg GAE/g.	[40]
Quinoa (peptides)	Isolation and characterization of bioactive peptides; molecular docking.	Chymotrypsin-derived bioactive peptides showed CI_50_ of 0.51 mg/mL (CEase) and 0.78 mg/mL (PL); 4 CEase-inhibitory and 12 PL-inhibitory peptides identified, suggesting natural antihypercholesterolemic potential.	[41]
Amaranth (bioactive films)	Development of films incorporating extracts/hydrolysates; functional testing.	Glycerol at 0.37–1.00% increased EB (12.19% to 2.20%), while 2% phenolic compounds significantly decreased EB and TS.	[46]
Quinoa (auxins/bioactivity)	Auxin analysis and functional evaluation.	14 new oxindoleacetate conjugates identified in quinoa seeds via UHPLC-QTOF-MS/MS and UHPLC-QOrbitrap-MS/MS using methanol/water and acetone/water.	[98]
Potato (conservation via coatings)	Storage assays with edible coatings and quality measurements.	Edible coatings significantly improved the chroma of red potatoes; F1 and F2 notable, while F1 and F4 increased anthocyanins at 3 months.	[55]
Sweet potato (peptides via sonication)	Ultrasound + enzymatic hydrolysis; peptide characterization.	Ultrasonic hydrolysis generated < 3 kDa peptides with high antioxidant activity, Fe^2+^ chelation, OH radical scavenging, and elevated ORAC values.	[99]
Tubers (encapsulation evaluation)	Encapsulation studies and functional assays	Encapsulation of sweet potato nodal segments with 4% alginate, 100 mM CaCl_2_, and ½ MS accelerated shoots, roots, growth, and genetic conservation.	[45]
Amaranth (protein) + cocoa pectin + phenolic extract	Coacervate complexes (AP: CP 2:1 and 5:1), phenolic extract (0–0.5% *w*/*v*), ζ-potential, FTIR, SEM.	ζ-potential near 0 mV (−1.8 to +0.9 mV); coacervation yield increased 35–48% depending on AP: CP ratio; antioxidant activity increased 20–45% with 0.5% PE; more porous structures confirmed by SEM.	[100]

Note. The details presented in the table are related to the matrix or species, method or approach, and the results obtained from experimental studies conducted between 2020 and 2025. ↑ indicates an increase, ↓ indicates a decrease. Abbreviations: BPE, bound polyphenols; IC_50_, half maximal inhibitory concentration; TPC, Total Phenolic Content; PCA, principal component analysis; PC1, Principal Component 1; PC2, Principal Component 2; OPLS-DA, Orthogonal Partial Least Squares Discriminant Analysis; CS, orange sweet potato flesh; ZS, purple sweet potato flesh; BS, white sweet potato flesh; AEC-TEO, alginate-based edible coating containing thyme essential oil; QTOF, Quadrupole Time-Of-Flight; QOrbitrap, Quadrupole Orbitrap.

## Data Availability

No new data were created or analyzed in this study. Data sharing is not applicable to this article.
